# Time-Course Analysis of Gene Expression During the *Saccharomyces cerevisiae* Hypoxic Response

**DOI:** 10.1534/g3.116.034991

**Published:** 2016-11-09

**Authors:** Nasrine Bendjilali, Samuel MacLeon, Gurmannat Kalra, Stephen D. Willis, A. K. M. Nawshad Hossian, Erica Avery, Olivia Wojtowicz, Mark J. Hickman

**Affiliations:** * Department of Mathematics, Rowan University, Glassboro, New Jersey 08028; † Department of Biological Sciences, Rowan University, Glassboro, New Jersey 08028; ‡ Department of Chemistry and Biochemistry, Rowan University, Glassboro, New Jersey 08028

**Keywords:** mRNA, transcriptome, anaerobic, signaling, metabolism

## Abstract

Many cells experience hypoxia, or low oxygen, and respond by dramatically altering gene expression. In the yeast *Saccharomyces cerevisia*e, genes that respond are required for many oxygen-dependent cellular processes, such as respiration, biosynthesis, and redox regulation. To more fully characterize the global response to hypoxia, we exposed yeast to hypoxic conditions, extracted RNA at different times, and performed RNA sequencing (RNA-seq) analysis. Time-course statistical analysis revealed hundreds of genes that changed expression by up to 550-fold. The genes responded with varying kinetics suggesting that multiple regulatory pathways are involved. We identified most known oxygen-regulated genes and also uncovered new regulated genes. Reverse transcription-quantitative PCR (RT-qPCR) analysis confirmed that the lysine methyltransferase *EFM6* and the recombinase *DMC1*, both conserved in humans, are indeed oxygen-responsive. Looking more broadly, oxygen-regulated genes participate in expected processes like respiration and lipid metabolism, but also in unexpected processes like amino acid and vitamin metabolism. Using principle component analysis, we discovered that the hypoxic response largely occurs during the first 2 hr and then a new steady-state expression state is achieved. Moreover, we show that the oxygen-dependent genes are not part of the previously described environmental stress response (ESR) consisting of genes that respond to diverse types of stress. While hypoxia appears to cause a transient stress, the hypoxic response is mostly characterized by a transition to a new state of gene expression. In summary, our results reveal that hypoxia causes widespread and complex changes in gene expression to prepare the cell to function with little or no oxygen.

Molecular oxygen (O_2_) plays a major role in the metabolism of many eukaryotic cells. Oxygen is not only required for aerobic respiration and for several biosynthetic reactions, but also influences the redox state and causes oxidative damage (Rosenfeld and Beauvoit 2003). The levels of oxygen vary in the environment. Hypoxia, or low oxygen, is experienced in certain tissues, diseases (*e.g.*, cancer), and specific environments (*e.g.*, deep water or soil). One way that cells cope with changing oxygen levels is by dramatically altering gene expression (Semenza 2011; Butler 2013; Ratcliffe 2013). In the yeast *Saccharomyces cerevisiae*, several microarray studies have found that the mRNA levels of hundreds of genes change in response to hypoxia (ter Linde *et al.* 1999; Becerra *et al.* 2002; Ter Linde and Steensma 2002; Kwast *et al.* 2002; Lai *et al.* 2005, 2006; Hickman and Winston 2007; Hickman *et al.* 2011). As might be expected, some responsive genes participate in oxygen-dependent cellular processes, such as aerobic respiration and the biosynthesis of unsaturated fatty acids (UFAs), heme, and ergosterol (the yeast functional equivalent of cholesterol). Other responsive genes do not directly participate in oxygen-dependent processes but may compensate for the lack of oxygen. For example, cell wall genes are induced during hypoxia, likely to compensate for the inability during hypoxia to synthesize membrane lipids that are important for membrane fluidity and permeability (Abramova *et al.* 2001a).

Many genes have been found to consistently respond to hypoxia. For example, several studies have found that *CYC1*, encoding cytochrome c, is downregulated, while *HEM13*, encoding a heme biosynthetic enzyme, is upregulated (Zhang and Hach 1999; Lombardía *et al.* 2000; Ter Linde and Steensma 2002; Hickman and Winston 2007; Butler 2013). However, other genes exhibit conflicting responses between studies. The *ERG5* ergosterol biosynthetic gene responds to hypoxia in only a subset of studies while *COX5B*, encoding a cytochrome c oxidase subunit, has been found to be either up- or downregulated (see Supplemental Material, Table S3). The conflicting results between studies indicate that the hypoxic response has not been clearly defined. Having an accurate picture of how gene expression changes during the hypoxic response is critical for understanding the metabolic changes and signaling events that occur during the transition from high to low oxygen levels.

In studying a gene expression response, it is informative to observe a time course (Storey *et al.* 2005), as opposed to two conditions such as aerobic and hypoxic. First, a time course captures the diverse kinetics of each gene’s expression, which is useful in characterizing complex responses that employ several signaling pathways and transcription factors. Second, transient expression changes, which may be important in the response, will be identified in a time course. Third, following expression level over the course of the response will allow one to determine whether the response is transient or a transition to a new steady-state. At the end of a transient response, gene expression should return to preresponse levels. In contrast, if a new steady-state is reached, then expression levels different from preresponse levels will be reached and maintained.

A time course is especially useful in characterizing the multifaceted hypoxic response in which the initial lack of oxygen immediately causes secondary effects such as depletion of metabolites (*e.g.*, heme, ergosterol, and UFAs) (Chellappa *et al.* 2001; Hickman *et al.* 2011), reduced flux through the electron transport chain (Guzy *et al.* 2007), and a change in growth rate (Burke 1997; Brauer *et al.* 2008). Each of these secondary effects acts as a stimulus, activating a unique signaling pathway that regulates gene expression; heme depletion signals to the Hap1 pathway (Hickman and Winston 2007) while sterol depletion activates the Upc2/Ecm22 pathway (Hickman *et al.* 2011). Furthermore, it has been suggested that some of the gene expression changes during hypoxia are due to a transient stress response (Lai *et al.* 2008). Approximately 900 genes in the yeast genome respond to diverse types of stress and have been deemed part of the ESR (Gasch *et al.* 2000). It is important to determine whether hypoxia activates the ESR genes and is thus considered a stress.

Growth of *S. cerevisiae* without oxygen requires exogenous ergosterol and UFAs, because these essential metabolites require oxygen for their biosynthesis (Rosenfeld and Beauvoit 2003). Thus, previous studies have included these metabolites when growing yeast without oxygen (ter Linde *et al.* 1999; Abramova *et al.* 2001b; Lai *et al.* 2006). However, we and others have found that some genes respond to hypoxia due to depletion of ergosterol and UFAs. Replenishing the metabolites reduces the effects of hypoxia (Hughes *et al.* 2005; Hickman *et al.* 2011). Therefore, in order to measure how yeast gene expression responds to natural hypoxia, it is important to measure the change in gene expression that occurs in the absence of oxygen without adding ergosterol and UFAs.

To measure global gene expression during the hypoxic response, we employed a recently developed technique known as RNA-seq (Wang *et al.* 2009; Anders and Huber 2010). This method uses next-generation sequencing to determine the relative abundance of each gene’s transcript. Compared to DNA microarray analysis, RNA-seq exhibits higher sensitivity (to detect less abundant transcripts), a greater dynamic range (to measure greater fold changes), and superior reproducibility (to accurately follow gene expression over time). Thus, RNA-seq will allow a more detailed characterization of the hypoxic response.

In this work, we have tracked the global gene expression response to hypoxia over 4 hr. Using principal component analysis (PCA), we discovered that the hypoxic response largely occurs during the first 2 hr and then a new steady-state expression state is achieved. Subsequent time-course statistical analyses identified 816 genes that change significantly over time in response to hypoxia, identifying most of the expected oxygen-regulated genes as well as genes that were not discovered previously. Three genes were verified by RT-qPCR to be oxygen-regulated. Our analysis identified genes that changed dramatically (2–550-fold) during the hypoxic response. The regulated genes were involved in expected (*e.g.*, respiration, lipid metabolism, and cell wall) and unexpected (*e.g.*, DNA and amino acid metabolism) cellular processes. Finally, we found that hypoxia causes a modest and transient stress response that is overshadowed by a more significant response that is unique to hypoxia.

## Materials and Methods

### Strain and growth conditions

The *S. cerevisiae* strain used is a *GAL2^+^* derivative of S288C containing a repaired *HAP1* allele (Hickman and Winston 2007), and is available upon request. Cells were grown at 30° in YPD (1% yeast extract, 2% peptone, and 2% glucose). For the hypoxia time course, cells were grown aerobically for at least four generations to midlog phase (1–2 × 10^7^ cells/ml). At time 0, a sample was taken, and then the cells were diluted in flasks so that they would reach midlog by the indicated time point. Hypoxia was achieved by continuously sparging flasks with ultrahigh-purity nitrogen gas at 3 L/min. Ergosterol and Tween 80 (source of UFAs) were not added to the media (Hickman *et al.* 2011). For each time point, 20 ml of cell culture was filtered using a 0.45 µm filter and microanalysis filter holder (Millipore), and the cells were snap frozen in liquid nitrogen. Removal from hypoxia and freezing took <30 sec, minimizing cell exposure to oxygen.

### RNA-seq and quality control filtering

Note that only one replicate of the hypoxia time course was used for RNA-seq analysis. RNA was prepared from frozen cells using mechanical disruption and the QIAGEN RNeasy kit. RNA quality was tested using the Bioanalyzer 2100 (Agilent). RNA concentration was determined using the Life Technologies Qubit, and RNA was diluted to 100 ng/µl for cDNA library preparation. The mRNA was enriched using oligo-dT capture and the cDNA library was prepared using the TruSeq RNA Sample Preparation Kit (Illumina) according to the manufacturer’s instructions. Eight RNA samples were barcoded, pooled, and sequenced in one lane using an Illumina HiSequation 2500 sequencer. For each sample, there were > 6.8 million reads, each at a length of 65 nucleotides. The reads were quality trimmed and then mapped to the *S. cerevisiae* S288C reference genome (SGD R64-1-1_20110203) using Tophat2 with default settings (Kim *et al.* 2013). At least 90% of reads in each sample successfully mapped. The number of reads mapping to each annotated feature was determined using HTSeq (Anders and Huber 2010), resulting in at least 4.98 million total reads per sample and an average of 697 reads per feature. The resulting FASTQ files and raw HTSeq data were deposited in NCBI’s Gene Expression Omnibus (Edgar *et al.* 2002) and are accessible through GEO Series accession number GSE85595. Genes with zero reads in all samples were removed (419 genes). To account for between-sample differences in sequencing depth, we used total-count normalization so that, for each sample, the total number of reads that maps to annotated features is equal, similar to calculating RPKM (Trapnell *et al.* 2012). Finally, the 24 *PAU* genes were removed because they are similar, and in some cases identical, in sequence, making them difficult to differentiate (Hickman *et al.* 2011). The normalized read count data and other information about each gene are included in Table S1.

### Experimental validation using RT-qPCR

Multiple biological replicates of the hypoxia time course were performed for RT-qPCR analysis. Total RNA was converted to cDNA using the BioRad iScript kit and poly-dT primers. The cDNA was subject to qPCR amplification using the BioRad iTaq-SYBR kit and the BioRad CFX96 Real Time System. Primer sequences are included in Table S2. Control qPCR reactions were performed on an equal amount of RNA that was not reverse transcribed, showing that there was no gDNA contamination.

### Time-course analyses

To identify genes that respond to hypoxia over time, we used two methods: DESeq2 and “AutoCor,” an algorithm developed here. DESeq2 was used to perform a likelihood ratio test (LRT in the DESeq2 package) (Love *et al.* 2014) that compares how well a gene’s count data fit a “full model” (with independent variables, like time) compared to a “reduced model” (without those variables). Our full model was a quadratic equation: *E_t_* = β_2_*t*^2^ + β_1_*t* + β_0_ where *E_t_* is normalized counts at time *t*, *t* is time, and each β is a coefficient. Our reduced model excluded time: *E_t_* = β_0_. In DESeq2, the full model was written ∼ time + I(time^2) and the reduced model was written ∼1. The rationale for this design was to test whether a gene’s expression fits a pattern of increase or decrease over the time points. In analyzing count data, DESeq2 estimates dispersion of each gene’s expression by taking into account the dispersion of genes expressed at similar levels. Since there were not biological replicates for each time point, all time points were combined as replicates to calculate dispersion. The dispersions of hypoxic-responsive genes will be overestimated but partially corrected by the lower dispersion of nonresponsive genes. The second method, AutoCor, is permutation-based and tests the importance of time point order. For each gene, the normalized count values in their original order were compared to all possible permutations (40,320). By using the actual normalized read count values, we did not make any assumptions about the distribution of the data. In the first step, the autocorrelation (lag = 1) (Box *et al.* 2008) for each gene is calculated. Autocorrelation is high when the values in a time series “persist” (*i.e.*, form a “smooth” curve). Then, for each gene, the autocorrelation was computed for each permuted time course. Finally, a one-sided p-value was calculated as the proportion of random autocorrelations that are greater than or equal to the original autocorrelation.

For the 2868 genes that each had a maximum fold-change over the entire hypoxic time course of ≥2, the p-values generated by DESeq2 or AutoCor were adjusted for multiple testing using the BH procedure (Benjamini and Hochberg 1995), and adjusted p ≤ 0.05 indicates significance. All statistical analyses, unless otherwise stated, were performed using R Studio (R Core Team 2015).

### PCA, Euclidian distance, clustering, and gene ontology (GO) analyses

All analyses in this section were performed on normalized read counts that were log2 transformed. PCA, a dimensionality reduction technique, was carried out in *R* by using the *prcomp* function (Q-mode, or singular value decomposition) on the matrix containing genes in columns and time points in rows (Hothorn and Everitt 2014). *R* outputs the PCA results into one variable (*e.g.*, *PC*) and resulting loadings are found in the matrix designated by *PC$rotation*. The Euclidian distance between samples was calculated in *R* using the *dist* function on the matrix containing genes in columns and time points in rows. A heatmap was generated using the resulting distance matrix with the *heatmap* function in *R*. Hierarchical clustering was performed using Cluster 3.0 (Eisen *et al.* 1998), with uncentered correlation and average linkage. A heatmap was generated from the resulting cluster using TreeView (Saldanha 2004). GO enrichment analysis was performed using GO Slim Mapper at SGD (Cherry *et al.* 2012).

### ESR data

All of the ESR gene expression data were obtained from the Gasch study (Gasch *et al.* 2000). The data presented in this paper are displayed in the following order: 37° heat shock (5, 15, 30, and 60 min); 20 min 37° heat shock (from 17, 21, 25, 29, 33, and 37°), 0.32 mM hydrogen peroxide (10, 20, 30, 40, 50, 60, 80, 100, 120, and 160 min); 1 mM menadione (10, 20, 30, 40, 50, 80, 105, 120, and 160 min); 2.5 mM DTT (15, 30, 60, 120, 240, and 480 min); 1.5 mM diamine (5, 10, 20, 30, 40, 50, 60, and 90 min); 1 M sorbitol (5, 15, 30, 45, 60, 90, and 120 min); amino acid starvation (0.5, 1, 2, 4, and 6 hr); nitrogen depletion (0.5, 1, 2, 4, 8, 12, 24, 48, 72, and 120 hr); diauxic shift (seven consecutive time points); YPD (2, 4, 6, 8, 10, 12, 24, 48, 72, and 120 hr); carbon source (ethanol, galactose, raffinose, sucrose, and fructose); and temperature (15, 17, 21, 25, 29, and 36°). A gene was considered to be part of the ESR if the gene was either originally called by the Gasch study or changed more than twofold in 10 or more of the 13 different treatments.

### Data availability

The GEO series accession number is GSE85595 (http://www.ncbi.nlm.nih.gov/geo/query/acc.cgi?acc=GSE85595).

## Results

### Quantifying mRNA levels during the switch to hypoxia

To study gene expression during the switch from aerobic to hypoxic growth, cells were quickly frozen after 0, 5, 10, 30, 60, 120, 180, and 240 min of hypoxia. The mRNA was isolated and subjected to RNA-seq analysis, as described in the *Materials and Methods*, generating the number of sequencing reads per gene. This analysis was highly reproducible, as shown by comparing gene read counts between 0 and 5 min (Figure S1). Low read counts are not reliable so, for each gene, we compared the median to the coefficient of variation of counts across time points (Figure S2). Indeed, genes with < 20 read counts showed high variability and thus all read counts <20 were set, or “floored,” to 20.

**Figure 1:**
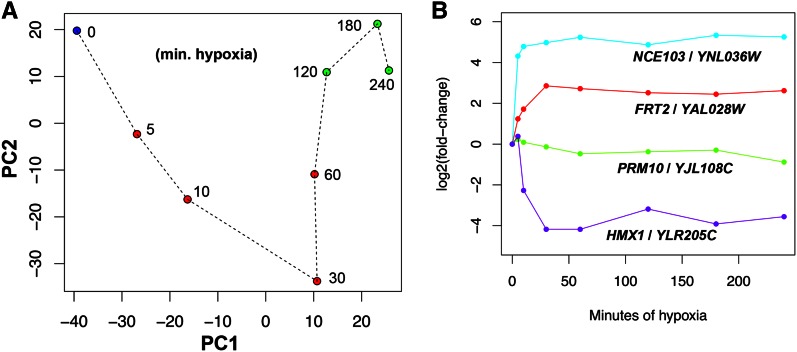
The gene expression response to hypoxia. (A) Principal component analysis to monitor global gene expression change during the switch to hypoxic growth. Log2 fold changes of normalized and floored count data were used. The aerobic time point (0 min) is in blue, middle time points (5, 10, 30, and 60 min) are in red, and late time points (120, 180, and 240 min) are in green. PC1 and PC2 captured 45 and 28%, respectively, of the variability in gene expression. (B) The hypoxic response of four different genes, as determined by our RNA-seq data. The read counts were normalized to time 0 and the ratios were log2 transformed. Though subtle, the *PRM10* gene shows a twofold change between the 5 and 240 min samples. min., minutes; RNA-seq, RNA sequencing.

Next, we examined the global change in gene expression during hypoxia by performing a PCA on all genes (Figure 1A). This analysis shows that there are large expression changes from 0 to 120 min. However, from 120 to 240 min, the changes are not as pronounced as shown by the closer proximity of the points. Thus, we conclude that most of the change occurs within the first 120 min of hypoxia and then a new steady-state of gene expression (*i.e.*, a hypoxic state) is reached. In addition, by observing expression at multiple times within 240 min, we were able to capture a large portion of the change that occurs during the switch from aerobic to hypoxic growth. One caveat to this analysis is that the first two components displayed in Figure 1A only comprise 73% of the variance and thus don’t represent the true distances between time points. Therefore, we calculated the Euclidian distances between each pair of time points and displayed the distance matrix as a heatmap (Figure S3A). This graph supports our conclusion that the last three time points (120–240 min) are highly similar to each other and less similar to earlier time points.

### Identifying time-dependent genes

**Figure 2:**
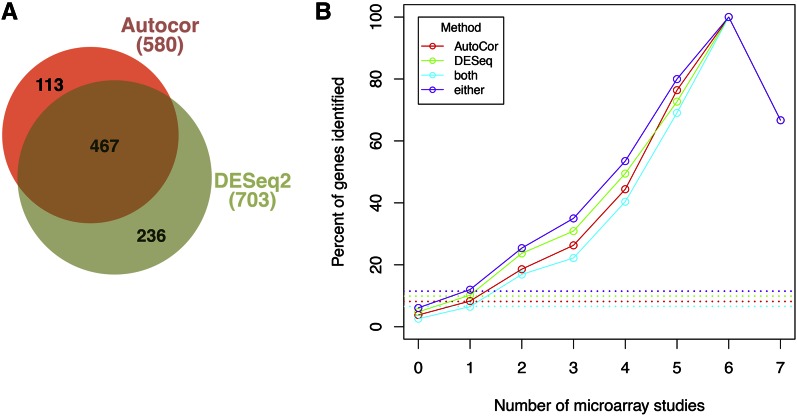
Identifying genes that respond to hypoxia in a time-dependent manner. (A) A Venn diagram showing that the two statistical methods used in this study detected largely overlapping sets of genes. The total number of genes detected by each method is shown in parentheses. (B) Our RNA-seq and statistical methods were more likely to identify well-known oxygen-regulated genes. The *x*-axis represents the number of independent microarray studies in which a gene was shown to be oxygen-regulated (as calculated in Table S3). The *y*-axis represents the percentage of those genes that were identified by the indicated method (both = AutoCor and DESeq2; either = AutoCor or DESeq2). The four curves overlap at *x* = 6 and *x* = 7. Each dotted line shows the percentage of genes in the genome that are detected by the indicated method. RNA-seq, RNA sequencing.

To identify genes that change expression as a function of time during hypoxia, we employed DESeq2 (Love *et al.* 2014) as described in the *Materials and Methods*. This method found 703 genes with a maximum fold-change of ≥2 during the time course. However, DESeq2 did not appear to identify all genes that respond to hypoxia, including three genes (*NCE103*, *FRT2*, and *PRM10*) that exhibited time-dependent changes here (Figure 1B) and were previously shown to be oxygen-regulated (Hickman and Winston 2007; Hickman *et al.* 2011). Thus, in order to capture these and other time-dependent genes not detected by DESeq2, we developed a permutation-based method, AutoCor, to test the importance of time (see *Materials and Methods*). AutoCor identifies genes with significantly high autocorrelation, signifying that expression changed smoothly over time. The AutoCor method identified 580 genes, including the *NCE103*, *FRT2*, and *PRM10* genes. We compared the AutoCor and DESeq2 methods using a Venn diagram (Figure 2A) and found that 467 genes were picked up by both methods (greater than the 61 expected if each method identified a random set of genes). In addition, the DESeq2 and AutoCor methods identified 236 and 113 unique genes, respectively, so that 816 genes showed significant time-dependence and thus were deemed oxygen-regulated.

Several microarray studies have been performed to find oxygen-regulated genes in *S. cerevisiae* (ter Linde *et al.* 1999; Ter Linde and Steensma 2002; Kwast *et al.* 2002; Becerra *et al.* 2002; Lai *et al.* 2005, 2006; Hickman and Winston 2007; Hickman *et al.* 2011) and we examined how well the current RNA-seq experiment detected the same genes (Figure 2B). First, we determined how many previous studies (0–7) found a gene to be oxygen-regulated (Table S3). Second, we calculated the percentage of these genes that were identified in the current RNA-seq experiment. Confirming that our methods were more likely to detect known oxygen-regulated genes, as the number of studies increases, the percentage identified also increases (Figure 2B, solid lines). In other words, we were more likely to identify a gene as oxygen-regulated the more times it had been observed previously. This relationship would not be expected if our analysis picked up a set of random genes (dotted lines). Moreover, genes that were not observed as oxygen-regulated previously (0 studies) were found here at a lower percentage than expected.

**Figure 3:**
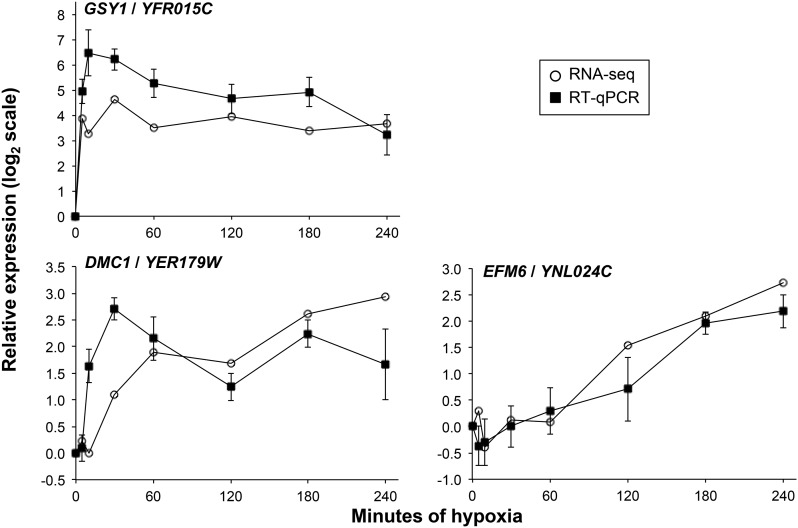
RT-qPCR analysis confirms RNA-seq results showing that *GSY1*, *DMC1*, and *EFM6* respond to hypoxia. The indicated gene was normalized to *FLC3*/*YGL139W*, which does not change during hypoxia (Table S1). qPCR was always performed on a no-RT control with negative results. Shown are means and SE of the mean for *n* = 3. Also shown is the relative expression using the RNA-seq read counts generated in the current study. RNA-seq, RNA sequencing; RT-qPCR, reverse transcription-quantitative polymerase chain reaction.

Our analysis so far has shown that we identified many of the known oxygen-regulated genes, but we wanted to know if our analysis missed any true oxygen-regulated genes. First, of the 11 genes found to be oxygen-regulated in six or seven previous microarray studies, we identified 10 here. Examination of the RNA-seq data shows that the one missed gene, *HMX1*, does indeed exhibit a rapid time-dependent response (Figure 1B). In fact, the AutoCor p-value was low (adjusted p=0.052), suggesting mild time-dependence. Second, we examined RNA-seq expression of the 11 genes identified as oxygen-regulated in five previous microarray studies but not here (Figure S4). This revealed two categories of genes. First, in agreement with our time-dependence analysis, six genes (*SFM1*, *FMP23*, *CYB2*, *FET4*, *HSP60*, and *PRY1*) showed little response. These genes do not respond to hypoxia in the strain or conditions used here. Second, five genes (*GSY1*, *HSP12*, *EMI2*, *MSC1*, and *SDS24*) changed by more than fourfold but were not detected as oxygen-regulated. These genes all exhibit rapid induction that may be difficult to detect with methods that test for time-dependence, because only one time point varies substantially from the others. For example, the *GSY1* gene showed a ∼16-fold induction within 5 min of hypoxia and remained highly expressed throughout the time course. We confirmed that this induction is reproducible using RT-qPCR (Figure 3). Thus, our statistical methods were not able to identify a subset of genes, but detected the vast majority of well-known oxygen-regulated genes (Figure 2B).

In addition, we discovered many new oxygen-regulated genes (statistics for all genes are shown in Table S1). Of the 4387 genes that were not observed as oxygen-regulated in any microarray study, we found 266 genes as oxygen-regulated here. To show that we were able to identify new oxygen-regulated genes, we used RT-qPCR to confirm that two of these genes, *DMC1* and *EFM6*, were indeed induced by hypoxia (Figure 3). Interestingly, *EFM6* (a putative S-adenosylmethionine-dependent lysine methyltransferase) and *DMC1* (a meiosis-specific recombinase required for double-strand break repair) both have orthologs in humans (*METTL21A* and *DMC1*, respectively).

We were surprised to identify such a large number of genes as oxygen-regulated, even though these genes had never or rarely been observed before. Of the 816 genes identified here, only 192 genes were detected as significantly oxygen-regulated in at least three previous microarray studies (Figure S5). This means that 624 genes were not consistently detected by microarrays. One hypothesis for why we identified these genes is that RNA-seq and our time-course analysis were able to identify genes with lower mRNA levels. Supporting this hypothesis, genes identified as regulated in this study were on average expressed lower than genes identified in microarray studies (Figure S6A). Further, the genes only identified in our study were on average expressed lower than genes identified both here and in microarray studies (Figure S6B). Thus, the sensitivity of RNA-seq and time-course analysis allowed us to uncover many additional oxygen-regulated genes.

### Description of the 816 time-dependent genes

**Figure 4:**
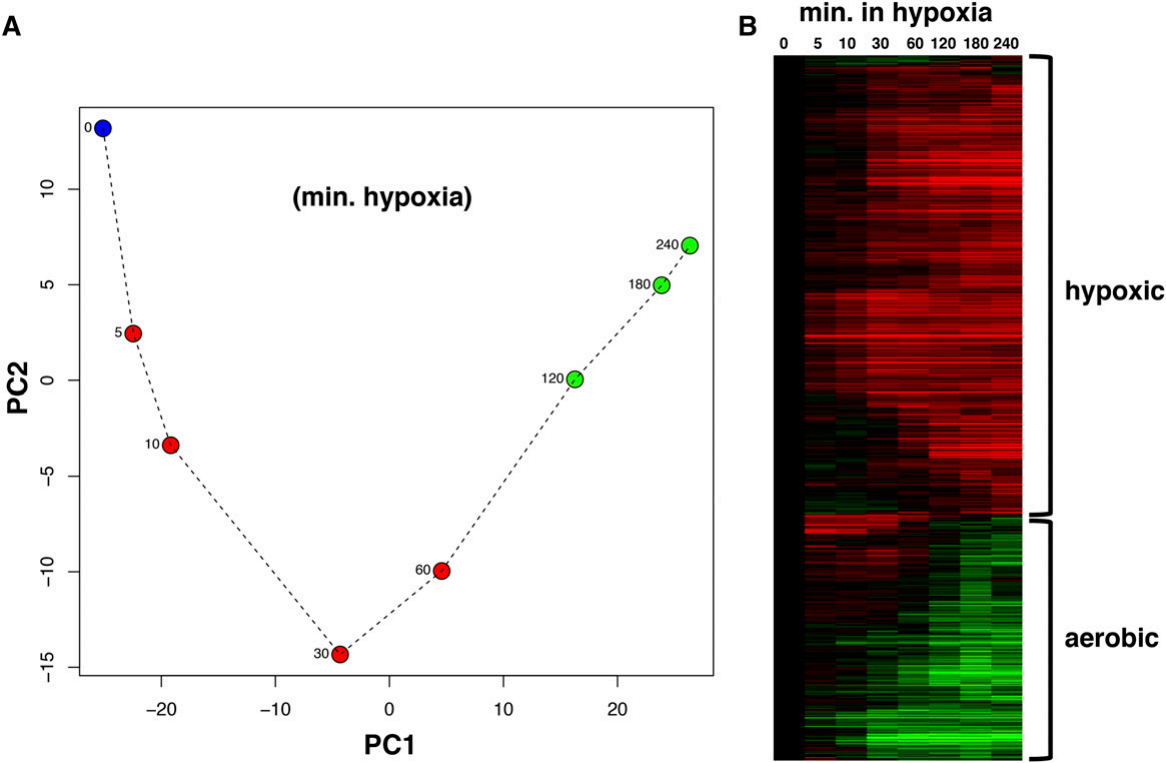
The hypoxic response of 816 genes that we identified as time-dependent. (A) Principal component analysis of the oxygen-regulated genes. This was performed and presented as in Figure 1A. PC1 and PC2 captured 75 and 14%, respectively, of the variability in gene expression. (B) Heatmap showing expression of the oxygen-regulated genes. Gene expression was normalized to time 0 (aerobic conditions) on a log2 scale. The matrix was subject to hierarchical clustering of the genes. The most intense red bar represents an increase by ∼550-fold while the most intense green bar represents a decrease by ∼52-fold. min., minutes.

In order to explore how the set of oxygen-regulated genes changes during the hypoxic response, we performed PCA (Figure 4A). Strikingly, the PCA pattern of this set is very similar to the pattern for all genes (Figure 1A), suggesting that the oxygen-regulated genes exhibit large changes in the first 2 hr followed by small changes. This is supported by a heatmap showing that the Euclidian distances between the last three time points is very small compared to their distances to earlier time points (Figure S3B). Thus, the oxygen-regulated genes appear to achieve a new “hypoxic” steady-state level of expression.

Next, we wanted to examine the kinetics of mRNA levels for the oxygen-regulated genes and thus displayed fold changes in a heatmap (Figure 4B). The 293 aerobic genes (repressed during hypoxia) and 523 hypoxic genes (induced during hypoxia) are indicated in the heatmap. The responsive genes exhibit varying kinetics, with changes occurring early or late during the response. For example, the well-known hypoxic gene *DAN1* (delayed anaerobic 1) does not change expression until 60 min, while the well-known aerobic gene *CYC1* (cytochrome c 1) responds within 10 min. Consistent with the PCA analyses, most expression changes occur from 0 to 120 min. For most genes, a new steady-state expression level is established by 120 min, as shown by minor expression changes between 120 and 240 min.

Gene expression levels changed dramatically during hypoxia, as determined by calculating the maximum fold change for each gene (Figure S7). The greatest increase in expression was almost 550-fold, while the greatest decrease was almost 52-fold. The top induced and top repressed genes are all well-known oxygen-regulated genes (Figure S7). These fold changes are greater than observed previously; for example, *DAN1*, which changed 550-fold here, was previously shown with microarray analysis to change 40-fold (Hickman *et al.* 2011). Not only does RNA-seq allow a more accurate quantitation of fold changes, but time-course analysis increases the chances of identifying the time when expression change is greatest.

### Cellular processes affected by hypoxia

The change from aerobic to hypoxic growth is expected to cause large changes in metabolism as well as alter expression of many genes involved in oxygen-dependent processes (Butler 2013). Previously, many studies have found that the expression of several cell wall, lipid, and respiration genes is changed during hypoxia (Burke 1997; ter Linde *et al.* 1999; Abramova *et al.* 2001a; Ter Linde and Steensma 2002; Lai *et al.* 2005). We used GO enrichment analysis (Cherry *et al.* 2012) to determine which processes were more than twofold enriched in the hypoxic or aerobic sets of genes (Table S4). As expected, the set of hypoxic genes was enriched for “lipid metabolic process” and the set of aerobic genes was enriched for “cellular respiration.” Even when examining the most highly-regulated genes (detected by both DESeq2 and AutoCor, as well as > fourfold change), many of the same processes were enriched (Table S4). Our analysis also revealed unexpected processes; for example “vitamin metabolic process” was enriched in the hypoxic set and “response to oxidative stress” was enriched in the aerobic set.

**Figure 5:**
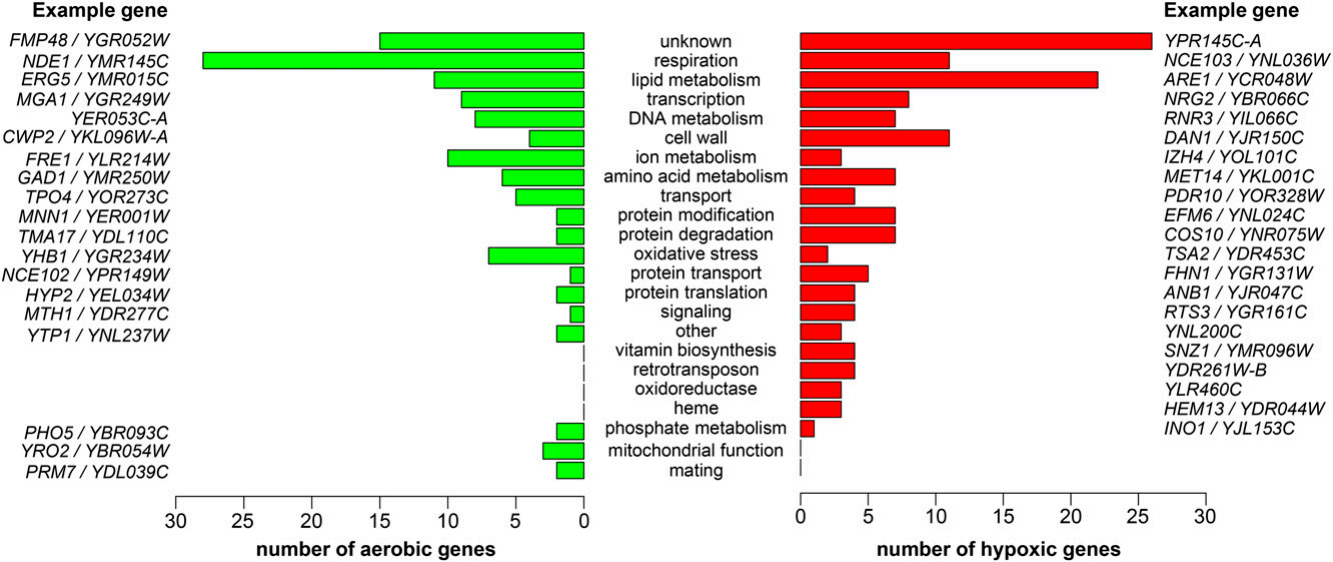
Cellular processes of the genes most highly affected by oxygen levels. Included are the 120 aerobic genes and 146 hypoxic genes that changed more than fourfold and are not considered dubious ORFs. The processes are in order by the number of genes in that process. For each function, example aerobic and hypoxic genes are included. ORFs, open reading frames.

Next, we manually assigned a process to each gene that changed more than fourfold (Table S1), to obtain a more complete picture of the processes that may be affected by oxygen levels (Figure 5). Many general aspects of this figure stand out. First, most of the processes contain both hypoxic and aerobic genes, indicating that the genes in a process are subject to both positive and negative regulation and thus that the process may be fine-tuned to perform better under hypoxia. One example is the set of genes implicated in oxidative stress. In response to hypoxia, seven such genes are downregulated while two genes are upregulated. One may predict that the production of reactive oxygen species decreases under hypoxia, but a different set of reactive species may be produced without oxygen (Guzy *et al.* 2007; Murphy 2009). Second, the most-populated process is “unknown,” which includes many widely conserved genes that have yet to be characterized. The fact that these are oxygen-regulated may give a clue to their functions. Third, genes in some of the processes (*e.g.*, respiration, lipid metabolism, heme, and cell wall) have been observed previously to be oxygen-regulated (Abramova *et al.* 2001b; Lai *et al.* 2005), further supporting a role for these processes in adapting to low oxygen.

Finally, our analysis identified many new processes containing oxygen-regulated genes; three such processes (transcription, DNA metabolism, and vitamin biosynthesis) will be discussed. First, we found that five transcription factor genes (*HAP1*, *HAP4*, *UPC2*, *MOT3*, and *ROX1*) not only regulate transcription of other genes in response to hypoxia (Butler 2013), but themselves change expression. This suggests that part of their regulation by oxygen levels involves regulation of their mRNA levels, perhaps by auto-regulation. Additionally, the fact that there are nine other transcription genes that respond to hypoxia illustrates the complexity in regulating gene expression during the hypoxic response. In the process “DNA metabolism,” most of the genes have been shown to be induced by DNA stress (Tkach *et al.* 2012), suggesting that hypoxia induces such stress. Three DNA metabolism genes specifically participate in nucleotide biosynthesis. The *ADE12* gene, encoding adenylosuccinate synthase, decreases expression during the switch to hypoxia, presumably causing cells to synthesize less AMP from IMP and thus decreasing the nucleotide pool for DNA and RNA synthesis. In contrast, the *RNR3* and *HUG1* genes are induced by hypoxia. Interestingly, *RNR3* encodes a nonessential subunit of the oxygen-dependent ribonucleotide reductase (RNR), responsible for creating dNTPs from NTPs. On the other hand, *HUG1* inhibits RNR activity by binding to a different subunit, Rnr2 (Meurisse *et al.* 2014). These results show that the multi-subunit RNR undergoes positive and negative regulation, which may fine-tune activity when oxygen levels change. In the process “vitamin biosynthesis,” hypoxia caused induction of four genes, all of which are directly or indirectly involved in the biosynthesis of B vitamins, cofactors in cell metabolism. *BIO2* encodes biotin (vitamin B_7_) synthase, *SNO1* and *SNZ1* are required for vitamin B_6_ production (Rodríguez-Navarro *et al.* 2002), and *THI22* is highly similar to thiamine (vitamin B_1_) biosynthetic enzymes (Llorente *et al.* 1999). The genes may be oxygen-regulated because many steps in vitamin biosynthesis are oxygen-sensitive or oxygen-dependent, and because these vitamins are known to be important for anaerobic respiration (Bartnicki-Garcia and Nickerson 1961; di Salvo *et al.* 2003; Lotierzo *et al.* 2005; Koenigsknecht and Downs 2010).

### The hypoxic response differs from the ESR

**Figure 6:**
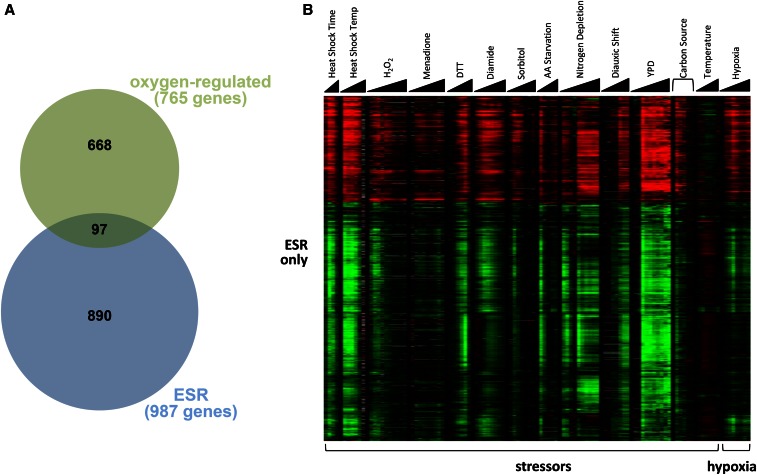
The hypoxic response is distinct from the ESR. (A) A Venn diagram shows that there is little overlap of the ESR and oxygen-regulated genes. The expected overlap, if these were two random sets, would be 124 genes. Only the 765 oxygen-regulated genes that were also examined in the ESR study were used in this analysis. (B) ESR genes respond modestly and transiently to hypoxia compared to the response to other stressors. The heatmap shows the expression of ESR genes that are not oxygen-regulated. The triangles under the conditions represent increasing concentrations or time. All conditions are listed in the *Materials and Methods*. The heatmaps in this figure and in Figure S8, Figure S9, and Figure S10 use the same gene expression scale to allow for cross-comparisons. The gray bars represent missing data from the ESR study. DTT, dithiothreitol; ESR, environmental stress response; H_2_O_2_, hydrogen peroxide; YPD, yeast extract peptone dextrose.

The ESR consists of hundreds of genes that respond to most types of stress, including DNA damage and oxidative stress (Gasch *et al.* 2000). We wanted to test whether hypoxia, which may be considered stressful, evokes the ESR. When comparing the 987 ESR genes and 816 oxygen-regulated genes, we found that only 97 of the oxygen-regulated genes were also ESR genes (Figure 6A), less than the 124 genes expected by chance. To compare the hypoxic and ESR responses in more detail, we examined the expression of four groups of genes: ESR only (Figure 6B), oxygen-regulated only (Figure S8), both oxygen-regulated and ESR (Figure S9), and regulated by neither oxygen nor ESR (Figure S10). First, as shown in Figure 6B, the ESR-only genes responded strongly to an array of stressors, as previously shown (Gasch *et al.* 2000), but responded modestly and transiently (with a peak of 30 min) to hypoxia before returning to prestress levels of expression. Interestingly, this modest response to hypoxia partially matched the response to stressors in that genes up- or downregulated by hypoxia were also up- or downregulated, respectively, by stressors. These results suggest that hypoxia causes a weak and transient stress response.

Second, the genes only regulated by oxygen exhibited a qualitatively and quantitatively distinct response to hypoxia compared to their response to stressors (Figure S8). For example, genes upregulated by hypoxia were not necessarily upregulated by stressors, but were either unchanged, upregulated, or downregulated. The genes that we have identified as oxygen-regulated clearly do not respond universally to stress, indicating that these genes comprise a unique set that is important for coping with low oxygen levels.

Third, only some genes that respond to both hypoxia and stressors exhibit a similar response to the two types of inducers (Figure S9). Many genes show opposing responses. As one notable example, *ANB1* (denoted by the arrow) is strongly upregulated by hypoxia but is slightly downregulated by stress. These results further support the idea that the gene expression response to hypoxia is distinct from the ESR.

Fourth, to confirm that we did not miss any responses to stress or hypoxia, we examined the expression of genes not found to be regulated by stress or by hypoxia (Figure S10). It is clear that these genes are not part of the ESR because they do not respond consistently to the different stressors. Also, they do not appear to respond significantly to hypoxia. Thus, the previous three groups of genes captured the majority of the response to stress or to hypoxia.

### PCA analysis of the entire hypoxic response

We identified two sets of genes (ESR and oxygen-regulated) as responding to hypoxia. To assess how these genes contribute to the overall change during the hypoxic response, we further analyzed the results from the PCA of all genes (shown in Figure 1A). Specifically, we observed the PC1 and PC2 loadings, which represent each gene’s contribution to the variance captured in each principal component (Figure S11A and Figure S11B). The genes with the greatest contribution to PC1 and PC2 are oxygen-regulated (blue and red points), showing that these genes contribute substantially to the hypoxic response. Interestingly, the oxygen-regulated genes contribute mainly to PC1 whereas ESR genes contribute mainly to PC2 (Figure S11A). A closer examination in Figure S11B shows that the border between these two sets of genes is not clearly defined, likely because some oxygen-regulated genes behave like ESR genes (*e.g.*, induced early with a peak at 30 min) and vice versa.

With the understanding of which genes contribute to PC1 and PC2 for all genes, we next focused on how each component changed during hypoxia. PC1 increased dramatically from 0 to 30 min, but then remained relatively constant at a new level for the remainder of the time course (Figure 1A), consistent with the idea that these genes (mainly oxygen-regulated) reach a new steady-state. PC2 decreased dramatically from 0 to 30 min, but then returned back to the starting level (Figure 1A), consistent with genes (mainly ESR) responding transiently and returning to their initial state. In contrast, when PCA was performed on the set of oxygen-regulated genes (Figure 4A), the resulting PC1 continued to increase until 180 min before stabilizing, much later than when considering all genes. PC2 followed a decrease–increase pattern similar to all-gene PCA (Figure 4A).

To understand this difference between all-gene PCA (Figure 1A) and oxygen-regulated PCA (Figure 4A), we compared which genes contributed to the variance in both analyses (Figure S12). Strikingly, the contributions of genes to PC1 were highly correlated between the two types of analyses (Figure S12A), showing that all-gene PC1 (describing 45% of variance) is highly similar to oxygen-regulated PC1 (describing 75% of variance). However, many genes were not included in this graph because they were not identified as oxygen-regulated (black and green data points in Figure S11A and Figure S11B), including the six genes described earlier (*HMX1*, *GSY1*, *HSP12*, *EMI2*, *MSC1*, and *SDS24*; triangles in Figure S11A and Figure S11B). The six genes all had high PC2 loadings, indicating that they may be ESR genes; indeed, three of them are. We reasoned that oxygen-regulated PC1 may exhibit a different pattern because these six genes are missing and thus adding them to the oxygen-regulated set would restore all-gene PC1 as in Figure 1A. Adding these six genes did not recreate all-gene PC1 (data not shown), but two other gene sets did: (1) the 1705 genes that are either oxygen-regulated or ESR (Figure S13A), or (2) the 885 genes that had all-gene PC1 and PC2 loadings > 0.01 (Figure S13B). Using the 168 genes with a PC threshold of ≥0.02 did not have nearly the same effect (Figure S13C). Taken together, the PCA analyses indicate that the entire gene expression response to hypoxia is comprised of a large, complex, and overlapping set of oxygen-regulated and ESR genes.

## Discussion

In this study, we have examined the global gene expression response to hypoxia. By observing changes in the cellular transcriptome at several time points spanning 4 hr, we were able to gain several insights into this response. First, a majority of the response occurs within the first 2 hr, leading to a new steady-state of expression. In this hypoxic state, the cell can presumably carry out essential processes without oxygen, provided that certain nutrients are available in the environment (Thomas *et al.* 1998). In addition, hypoxia causes the ESR genes to respond transiently with a peak at 30 min, suggesting that the transition from the aerobic to hypoxic state is stressful, due to several possible factors (*e.g.*, nitric oxide, metabolite depletion, membrane disruption, or low energy) (Lai *et al.* 2005; Poyton *et al.* 2009; Hickman *et al.* 2011). However, the ESR is a minor component of the hypoxic response, which is mainly comprised of a unique set of genes. Furthermore, genes respond to hypoxia with varying kinetics, reflecting the complexity of the response. One reason for this complexity is that hypoxia causes multiple secondary events (*e.g.*, decreased energy production, depletion of multiple metabolites, and change in redox state) (Poyton *et al.* 2009; Hickman *et al.* 2011; Butler 2013). These events likely occur at different rates following oxygen withdrawal and each serve as a stimulus for a different signaling pathway. Indeed, several signaling pathways are known to mediate the response to hypoxia, including Hap1/Rox1/Mot3 (Sertil 2003), Hog1/Upc2/Ecm22 (Hickman *et al.* 2011), Hap2/Hap3/Hap4/Hap5 (Ramil *et al.* 2000), Mga2 (Jiang *et al.* 2002), and the mitochondrion (Poyton *et al.* 2009). Now that we have described the genes and kinetics of the hypoxic response, it will be important to establish the role of each signaling pathway in regulating expression changes.

In this study, we measured transcript levels using RNA-seq, a method that is becoming the standard for measuring global gene expression (Wang *et al.* 2009; Ellahi *et al.* 2015). RNA-seq has three features that aided our ability to detect time-dependent changes. First, RNA-seq is extremely sensitive and thus, with sufficient sequencing depth, is able to detect low abundance transcripts. Indeed, genes that we identified to be oxygen-regulated exhibited lower expression on average than those detected by microarray experiments. Second, RNA-seq has a wide dynamic range; this feature contributed to our ability to detect up to 550-fold changes. Third, RNA-seq analysis is highly reproducible. We found that there was a high correlation between all of the samples, especially between the two samples predicted to be the most related (0 and 5 min).

To identify genes that respond to hypoxia, we used two statistical methods to examine the time-dependence of each gene’s read counts over the time course. This analysis revealed that 816 genes showed significant time-dependence by either the AutoCor or DESeq2 method. We compared our results to previous hypoxia microarray studies and found that we identified most of the well-known oxygen-regulated genes (Figure 2B). In addition, we discovered many new oxygen-regulated genes. For example, two genes, *DMC1* and *EFM6*, were identified here but not in previous studies, and also showed oxygen-regulation in follow-up RT-qPCR analysis (Figure 3). However, our analysis of time-dependence did not detect all oxygen-regulated genes, especially those with rapid kinetics (Figure S4). For example, *GSY1* was seen in five previous microarray studies but was not identified by our statistical methods. The RNA-seq data shows that *GSY1* is rapidly induced by hypoxia, and this is confirmed by RT-qPCR (Figure 3). To more accurately detect early expression changes, it will be necessary to collect additional RNA samples immediately before and after oxygen withdrawal.

The set of genes identified here as oxygen-regulated differed from what was found in other microarray studies (ter Linde *et al.* 1999; Ter Linde and Steensma 2002; Kwast *et al.* 2002; Becerra *et al.* 2002; Lai *et al.* 2005, 2006; Hickman and Winston 2007; Hickman *et al.* 2011). Some genes, like *DMC1* and *EFM6*, were only found here to be oxygen-regulated, while other genes, like *CYB2* and *HSP60*, were only found in other studies. One explanation is that the details of our experiments differed from previous studies in many important ways. First, to make sure that we captured the complete hypoxic response, we used an S288C-derived strain which contains a repaired *HAP1* allele (Hickman and Winston 2007) and is wild-type for other known oxygen-dependent regulatory factors (*ROX1*, *MOT3*, *UPC2*, *ECM22*, *MGA2*, HAP2/HAP3/HAP4/HAP5, and *HOG1*). Second, hypoxia was established by continuously sparging flasks with ultrahigh-purity N_2_, which lowers the dissolved [O_2_] immediately with reproducible kinetics (Lai *et al.* 2006). Third, ergosterol and UFAs were not added to the hypoxic cultures. Many studies add these metabolites because they are required for long-term anaerobic growth (Shianna *et al.* 2001). However, part of the hypoxic gene expression response is caused by depletion of metabolites (including heme, ergosterol, and UFAs) (Chellappa *et al.* 2001; Davies and Rine 2006; Hickman and Winston 2007; Hickman *et al.* 2011); thus, adding them may dampen or eliminate the response.

GO enrichment analysis revealed that many expected pathways (*e.g.*, respiration and lipid metabolism) respond to hypoxia. In order to obtain a more complete picture of the cellular processes affected, we grouped the most responsive genes into several manually-constructed categories (Figure 5 and Table S1). The results verified that the processes “respiration” and “lipid metabolism” contained a high number of responsive genes. In addition, our results revealed processes (*e.g.*, vitamin biosynthesis and metabolism of amino acids, DNA, and proteins) that have not been observed previously. Interestingly, many processes contained both up- and downregulated genes, suggesting that these processes are not simply activated or repressed, but are rewired to function optimally in hypoxia. Many regulated processes clearly require oxygen or are affected by oxygen levels, as discussed throughout this study. However, it is not clear how some of the processes are linked to oxygen levels. For example, there is not an obvious link between amino acids and oxygen; molecular oxygen does not appear to be required for amino acid biosynthesis. Despite this, exogenous amino acids can stimulate anaerobic growth of *S. cerevisiae* (Thomas *et al.* 1998), indicating that amino acid levels are limiting. Further studies are needed to determine the role of oxygen in each of the oxygen-regulated processes.

In conclusion, time-course analysis has shown that hypoxia causes widespread and complex changes in gene expression. Such analysis allowed us to observe not only a transient stress response, but also larger hypoxia-specific changes as the cell transitions from aerobic to hypoxic growth. The next challenge will be to delineate all of the regulatory pathways that mediate these changes.

## Supplementary Material

Supplemental material is available online at www.g3journal.org/lookup/suppl/doi:10.1534/g3.116.034991/-/DC1.
