# Supplementary material for: Time-Course Analysis of Gene Expression During the Saccharomyces cerevisiae Hypoxic Response
Source: G3 (Bethesda). 2016 Nov 9;7(1):221–31. doi: 10.1534/g3.116.034991 (PMC5217111; doi:10.1534/g3.116.034991)
Supplement: Supplementary file 1 [file 221FigureS1.pdf]

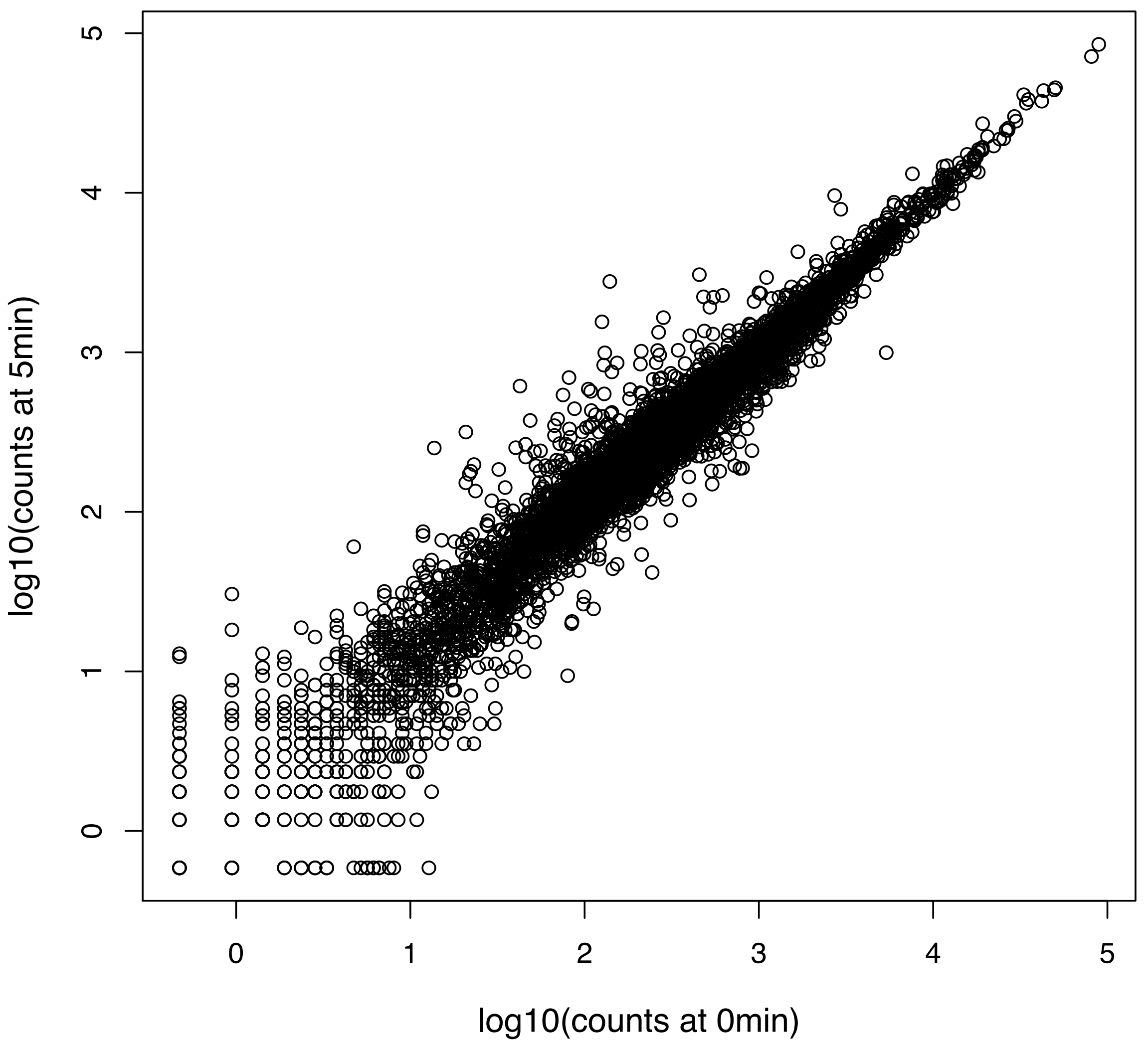

Figure S1. Reproducibility of RNA-seq, comparing number of counts between the 0- and 5- minute samples. Pearson correlation = 0.991. The lowest Pearson correlation when comparing all 8 time points is 0.866 (0- vs. 240-minute).
