# Supplementary material for: Time-Course Analysis of Gene Expression During the Saccharomyces cerevisiae Hypoxic Response
Source: G3 (Bethesda). 2016 Nov 9;7(1):221–31. doi: 10.1534/g3.116.034991 (PMC5217111; doi:10.1534/g3.116.034991)
Supplement: Supplementary file 2 [file 221FigureS2.pdf]

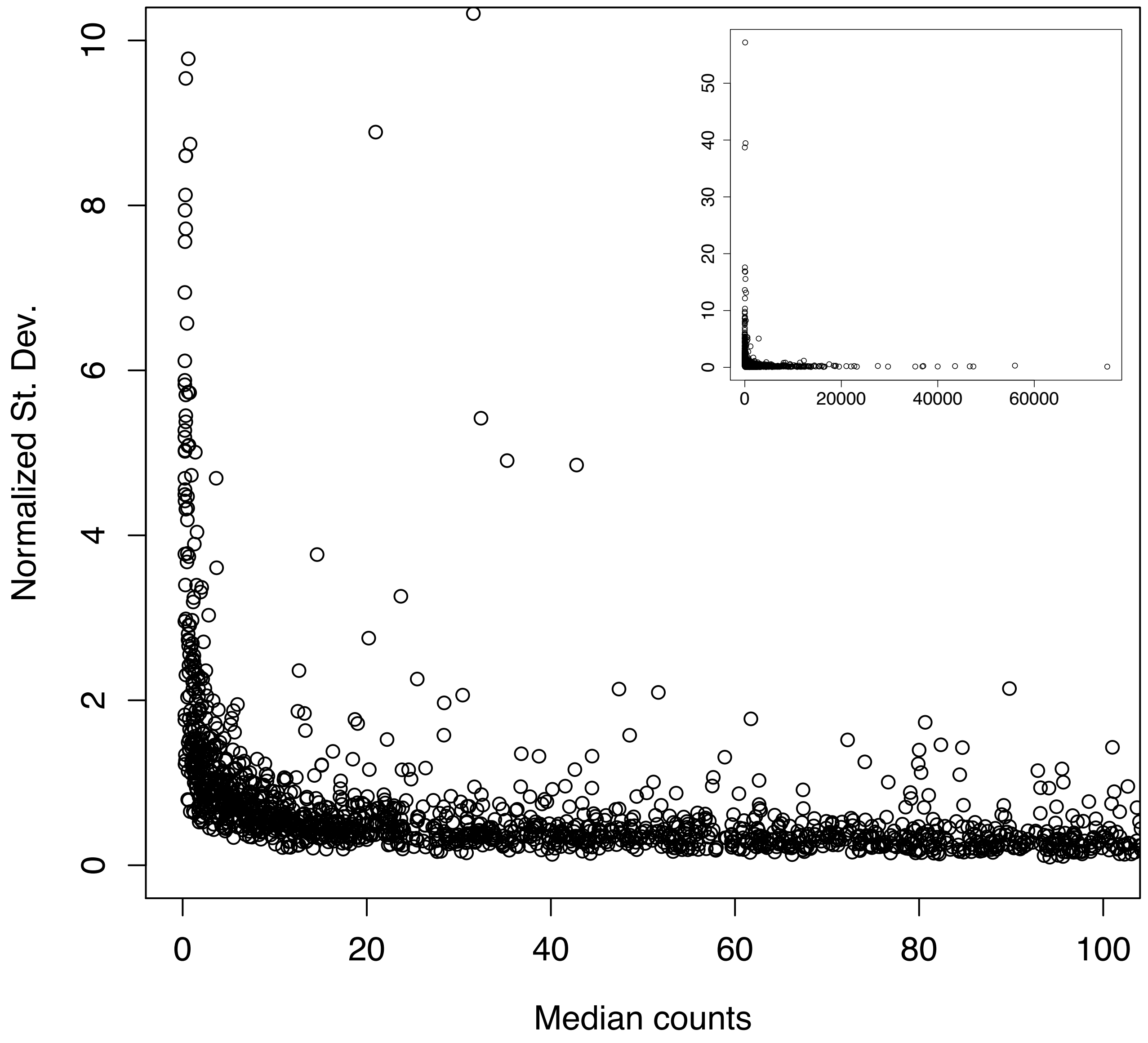

Figure S2. Determining the “floor” of read counts, or the minimum reliable read count. The median count and normalized standard deviation (sd/mean) for each gene was calculated across the eight time points. As the read count decreases below 20, the standard deviation increases dramatically, suggesting high technical variation. The larger graph presents 0-100 read counts, whereas the inset presents all data.
