# Supplementary material for: Time-Course Analysis of Gene Expression During the Saccharomyces cerevisiae Hypoxic Response
Source: G3 (Bethesda). 2016 Nov 9;7(1):221–31. doi: 10.1534/g3.116.034991 (PMC5217111; doi:10.1534/g3.116.034991)
Supplement: Supplementary file 3 [file 221FigureS3.pdf]

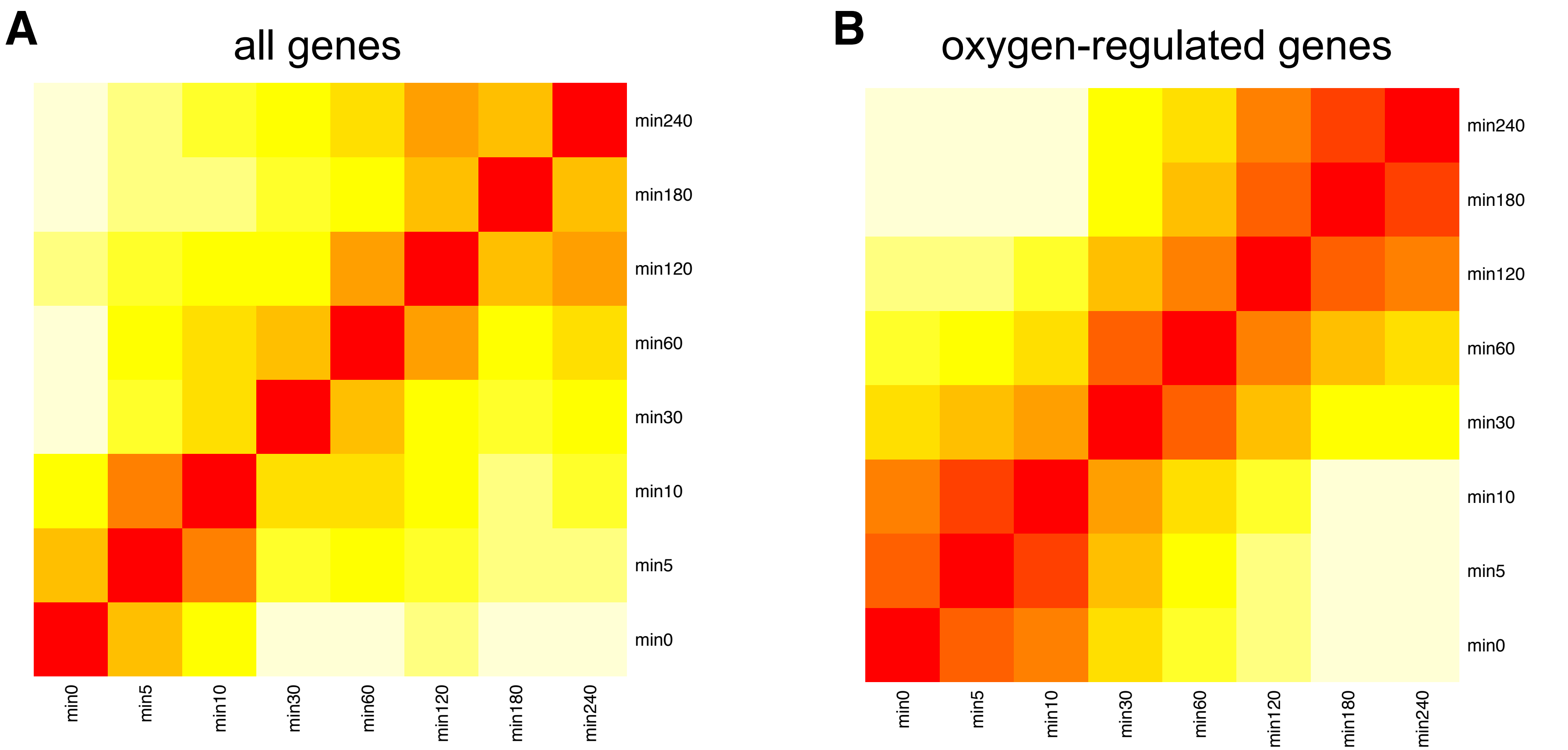

Figure S3. Heatmaps representing the Euclidian distance between samples. The log_2 transformed normalized expression values were input into the *dist* function in R. The darker colors indicate more closely-related samples, with red indicating identity. **(A)** Euclidian distance was determined between each pair of samples using expression of all genes. **(B)** Euclidian distance was determined between each pair of samples using expression of only the 816 oxygen-regulated genes.
