# Supplementary material for: Time-Course Analysis of Gene Expression During the Saccharomyces cerevisiae Hypoxic Response
Source: G3 (Bethesda). 2016 Nov 9;7(1):221–31. doi: 10.1534/g3.116.034991 (PMC5217111; doi:10.1534/g3.116.034991)
Supplement: Supplementary file 4 [file 221FigureS4.pdf]

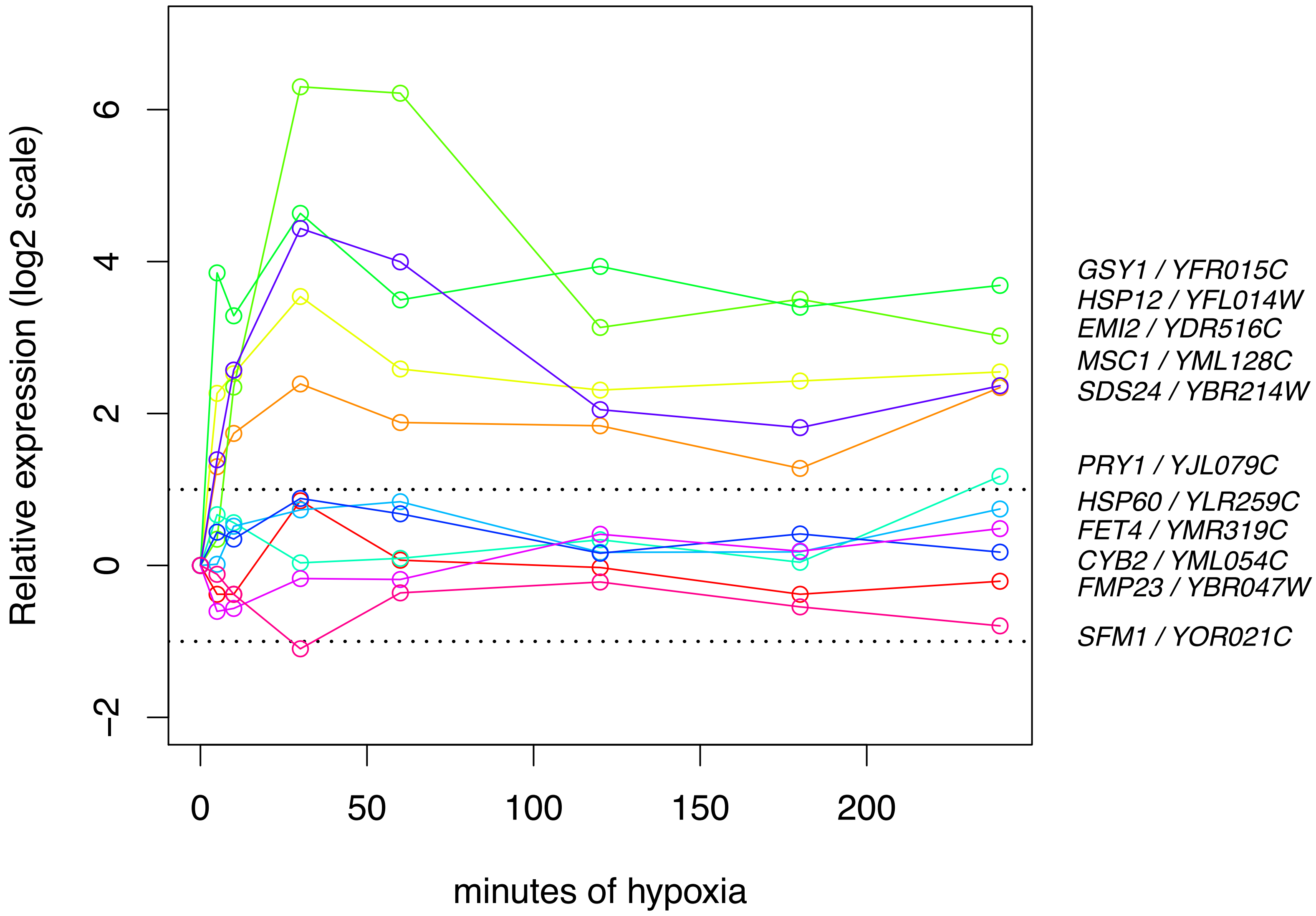

Figure S4. RNA-seq expression of the 11 genes identified as oxygen-regulated previously (observed in 5 previous microarray studies) but not identified here.
