# Supplementary material for: Time-Course Analysis of Gene Expression During the Saccharomyces cerevisiae Hypoxic Response
Source: G3 (Bethesda). 2016 Nov 9;7(1):221–31. doi: 10.1534/g3.116.034991 (PMC5217111; doi:10.1534/g3.116.034991)
Supplement: Supplementary file 5 [file 221FigureS5.pdf]

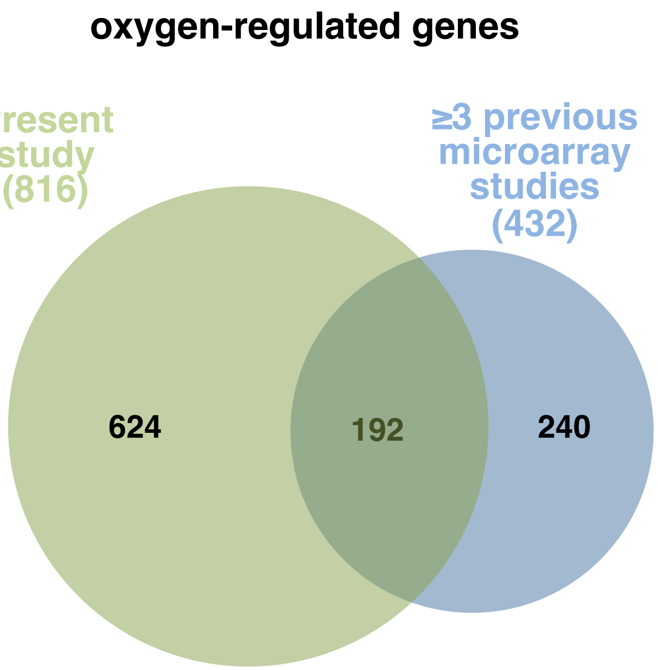

Figure S5. Venn diagram showing overlap of genes identified here by either one of the methods and genes identified in 3 or more previous microarrays. The overlap was 192 genes, but only 49 genes were expected to overlap by chance.
