# Supplementary material for: Time-Course Analysis of Gene Expression During the Saccharomyces cerevisiae Hypoxic Response
Source: G3 (Bethesda). 2016 Nov 9;7(1):221–31. doi: 10.1534/g3.116.034991 (PMC5217111; doi:10.1534/g3.116.034991)
Supplement: Supplementary file 6 [file 221FigureS6.pdf]

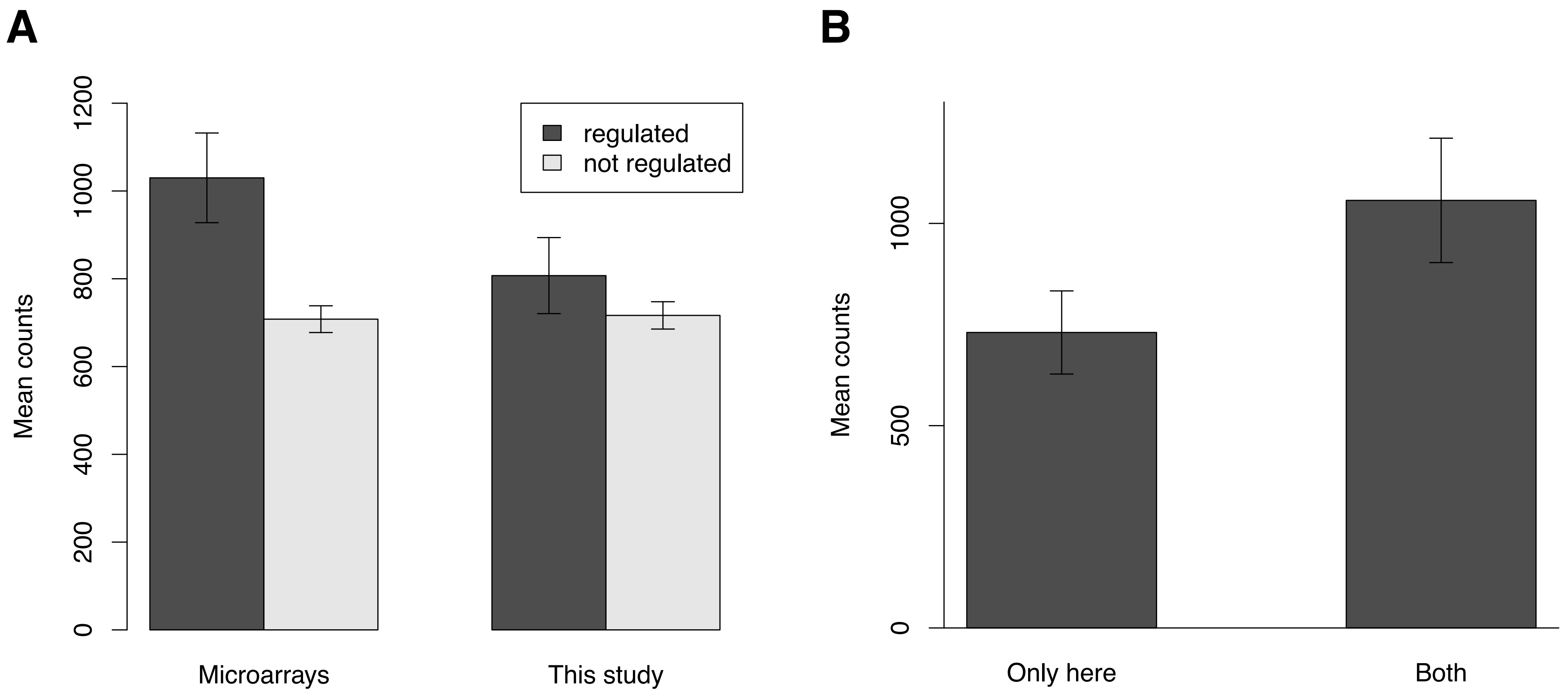

Figure S6. Genes identified as oxygen-regulated in the current RNA-seq study exhibited a lower expression level than genes identified in microarray studies. Mean counts were calculated from our RNA-seq normalized count data for each gene across the eight time points. A gene was determined to be detected by microarrays if observed in 3 or more microarray studies. Error bars represent the SEM. **(A)** Genes identified in microarrays were more highly expressed than genes identified in the current study. The expression of genes not regulated were the same between the two methods. **(B)** The genes identified as oxygen-regulated in only the current study (and not in microarray studies) exhibited a lower expression level than genes identified in both the current study and previous microarray studies.
