# Supplementary material for: Time-Course Analysis of Gene Expression During the Saccharomyces cerevisiae Hypoxic Response
Source: G3 (Bethesda). 2016 Nov 9;7(1):221–31. doi: 10.1534/g3.116.034991 (PMC5217111; doi:10.1534/g3.116.034991)
Supplement: Supplementary file 7 [file 221FigureS7.pdf]

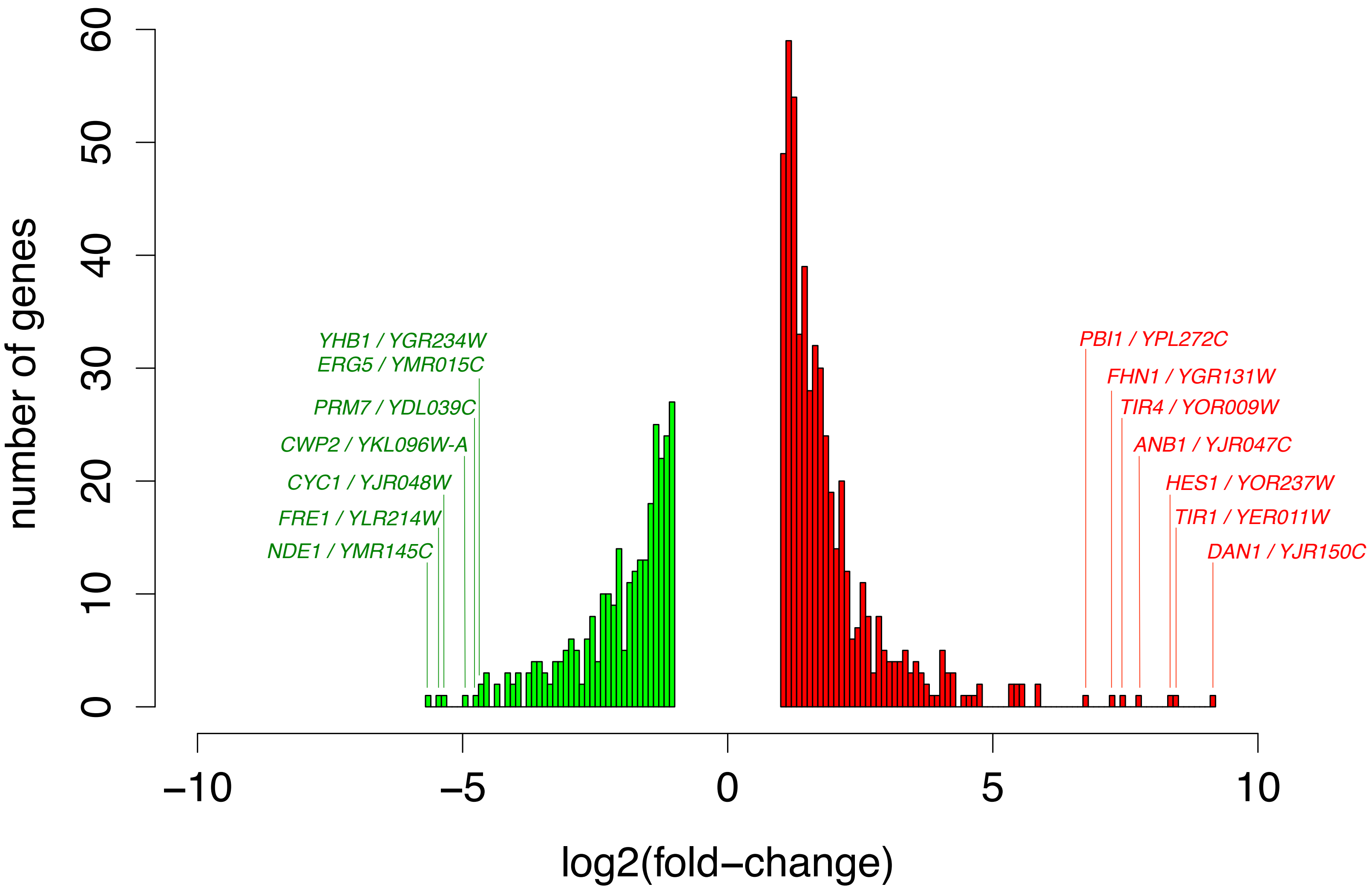

Figure S7. Wide distribution of fold-changes of the 816 oxygen-regulated genes identified by AutoCor or DESeq. The x-axis of this histogram represents the maximum fold change observed for a gene in the hypoxia time course. The genes with the lowest and highest fold changes are labeled. Other regulated genes are listed and described in Table S1.
