# Supplementary material for: Time-Course Analysis of Gene Expression During the Saccharomyces cerevisiae Hypoxic Response
Source: G3 (Bethesda). 2016 Nov 9;7(1):221–31. doi: 10.1534/g3.116.034991 (PMC5217111; doi:10.1534/g3.116.034991)
Supplement: Supplementary file 8 [file 221FigureS8.pdf]

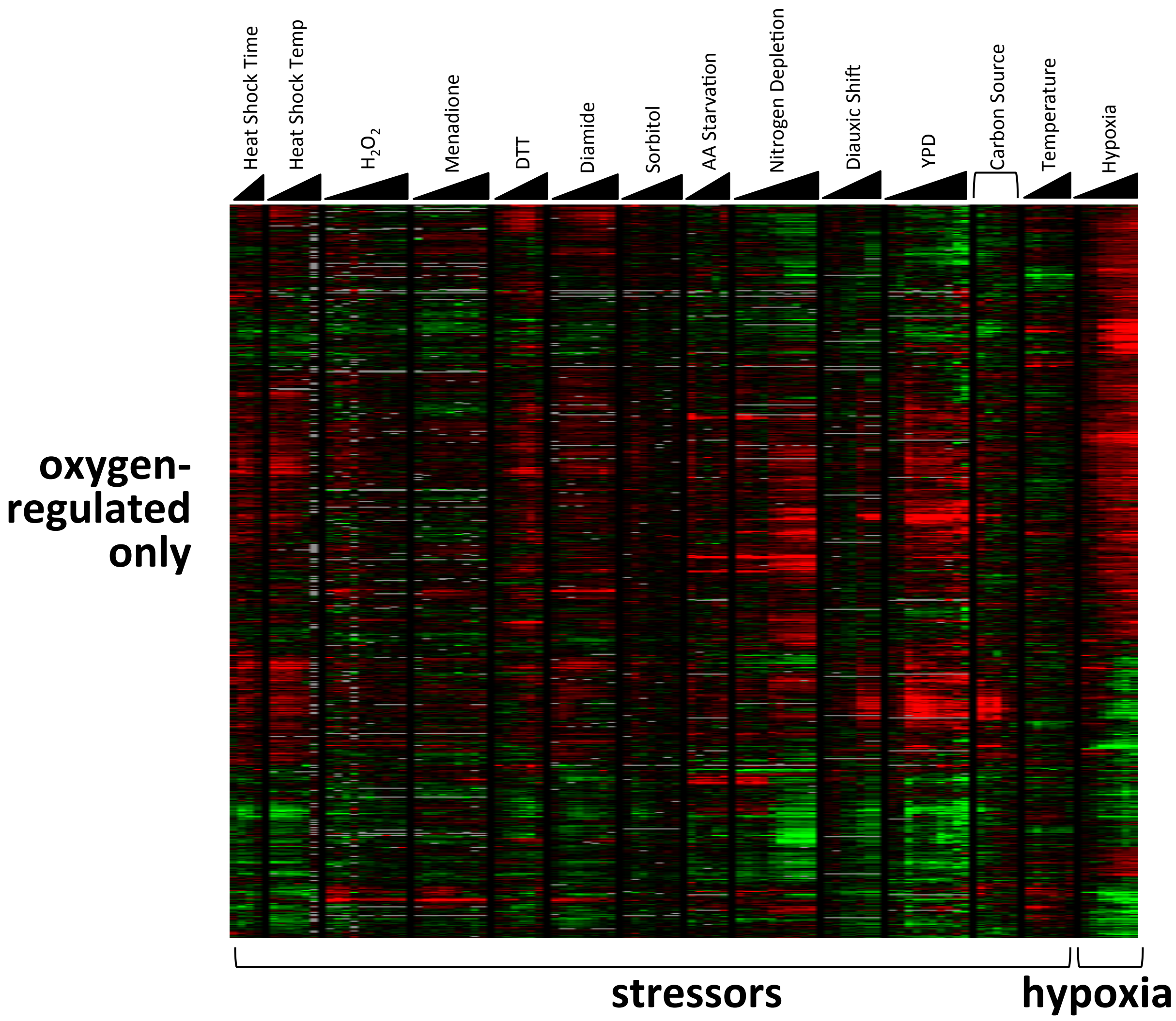

Figure S8. A heatmap shows that the hypoxic response is distinct from the response to stress. Shown are the genes that are oxygen-regulated but not part of the ESR.
