# Supplementary material for: Time-Course Analysis of Gene Expression During the Saccharomyces cerevisiae Hypoxic Response
Source: G3 (Bethesda). 2016 Nov 9;7(1):221–31. doi: 10.1534/g3.116.034991 (PMC5217111; doi:10.1534/g3.116.034991)
Supplement: Supplementary file 9 [file 221FigureS9.pdf]

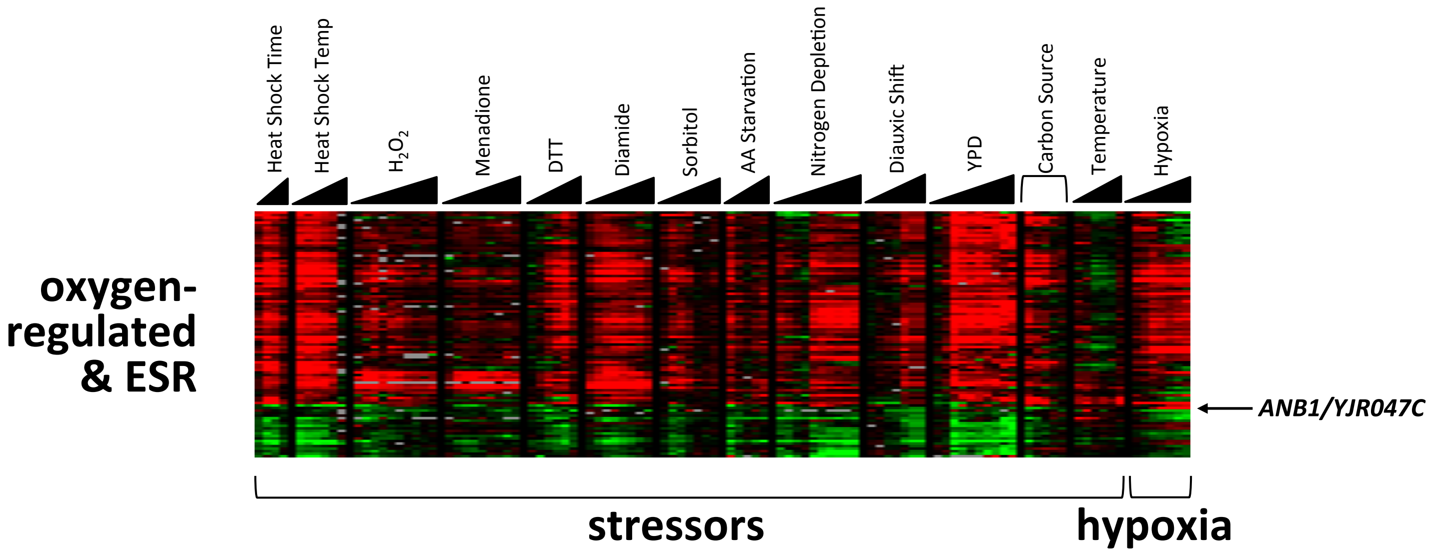

Figure S9. A heatmap shows that some, but not all, genes respond to both hypoxia and stress. Shown are the genes that are both oxygen-regulated and part of the ESR. The *ANB1* gene is indicated because it is induced by hypoxia but responds very differently to stress.
