# Supplementary material for: Time-Course Analysis of Gene Expression During the Saccharomyces cerevisiae Hypoxic Response
Source: G3 (Bethesda). 2016 Nov 9;7(1):221–31. doi: 10.1534/g3.116.034991 (PMC5217111; doi:10.1534/g3.116.034991)
Supplement: Supplementary file 11 [file 221FigureS11.pdf]

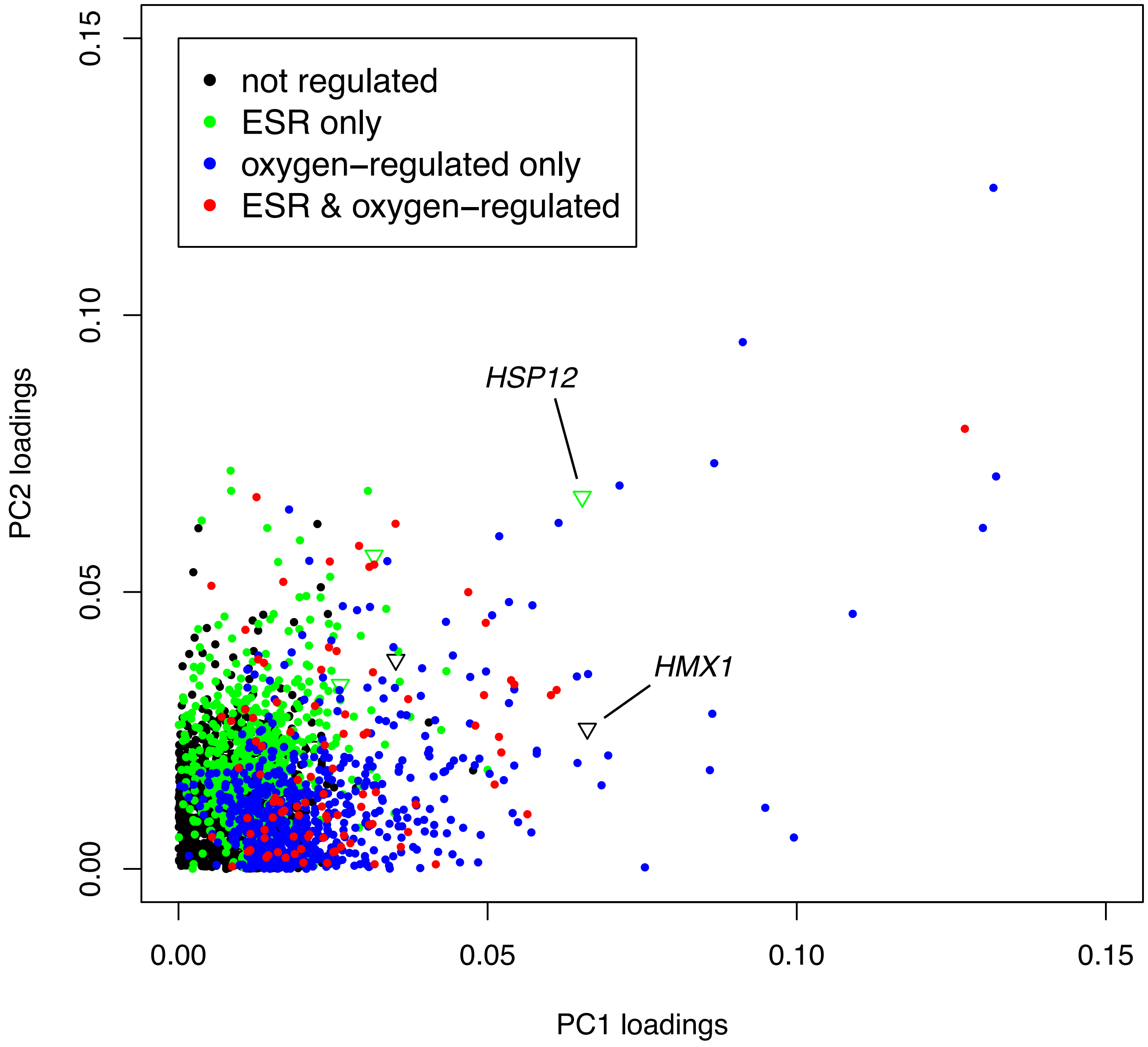

Figure S11A. The PC1 and PC2 loadings for all genes following PCA. Each data point is a gene. The six triangles represent genes that are discussed in the text and the triangles are color coded as in the legend.

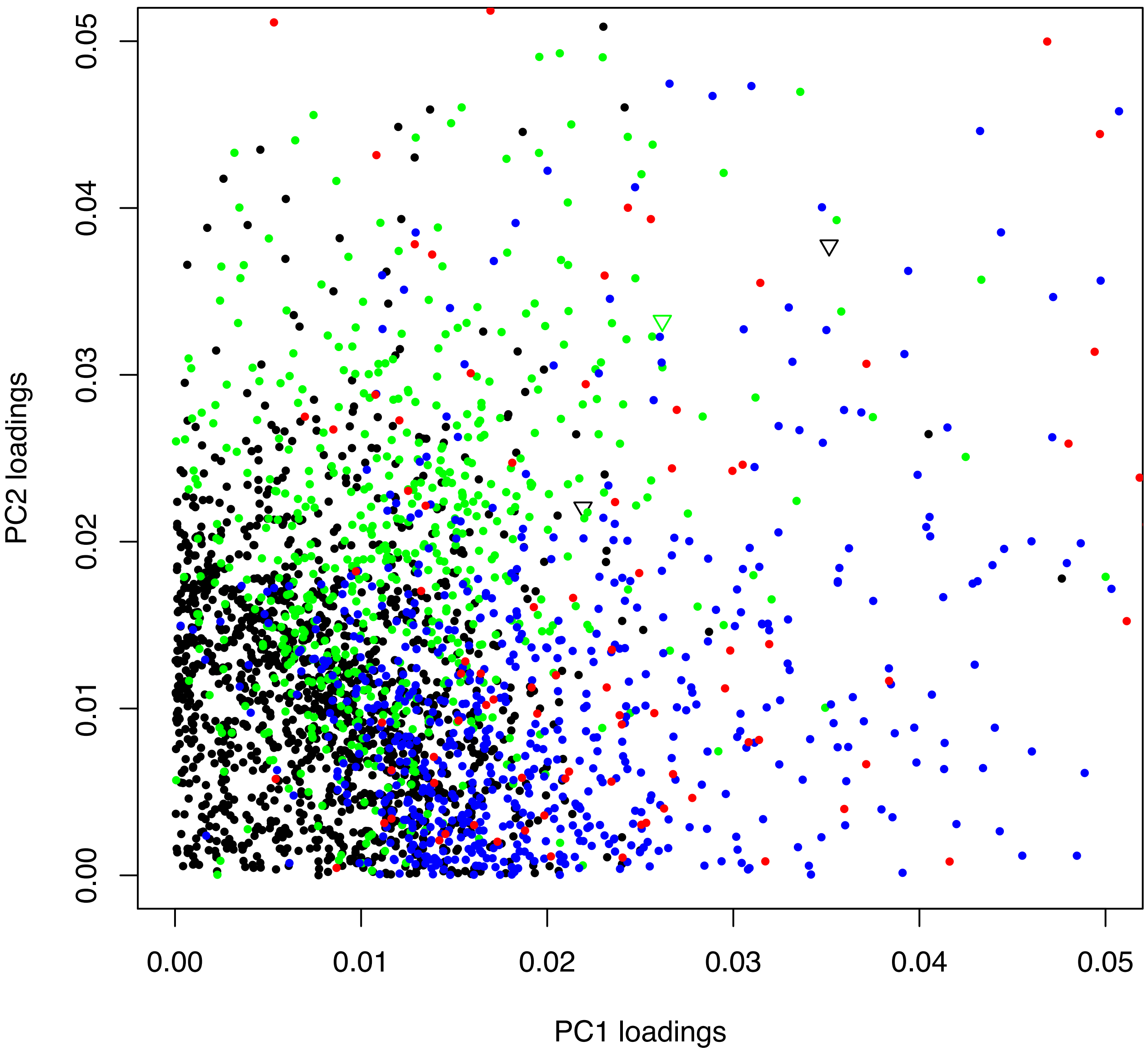

Figure S11B. This figure displays a zoomed-in version of the data in Figure S11A. Each axis scale is 0 to 0.05.
