# Supplementary material for: Time-Course Analysis of Gene Expression During the Saccharomyces cerevisiae Hypoxic Response
Source: G3 (Bethesda). 2016 Nov 9;7(1):221–31. doi: 10.1534/g3.116.034991 (PMC5217111; doi:10.1534/g3.116.034991)
Supplement: Supplementary file 12 [file 221FigureS12.pdf]

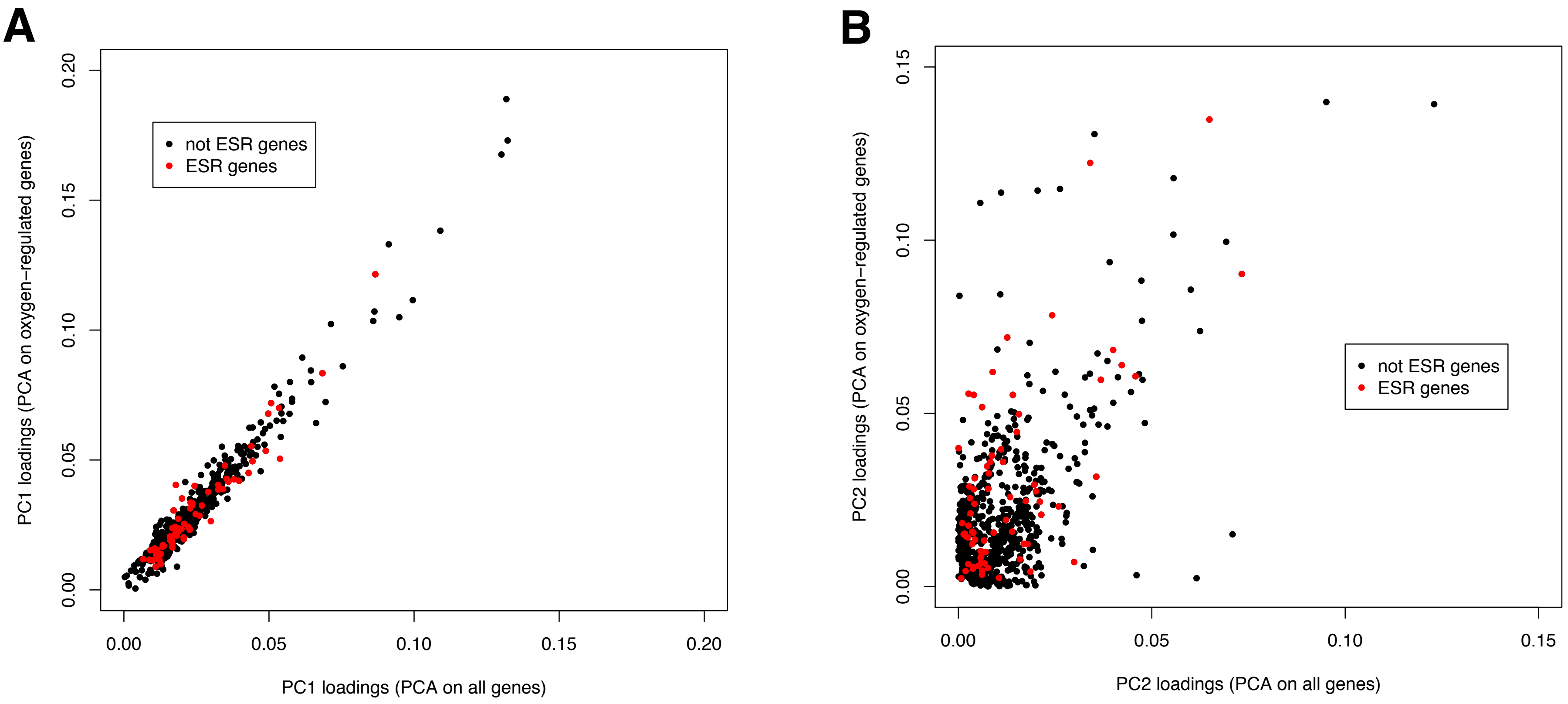

Figure S12. Comparison of the loadings between all-gene PCA (x-axes) and oxygen-regulated-gene PCA (y-axes). **(A)** PC1 loadings for each gene was compared between the two PCA analyses. **(B)** PC2 loadings for each gene was compared between the two PCA analyses.
