# Supplementary material for: Time-Course Analysis of Gene Expression During the Saccharomyces cerevisiae Hypoxic Response
Source: G3 (Bethesda). 2016 Nov 9;7(1):221–31. doi: 10.1534/g3.116.034991 (PMC5217111; doi:10.1534/g3.116.034991)
Supplement: Supplementary file 13 [file 221FigureS13.pdf]

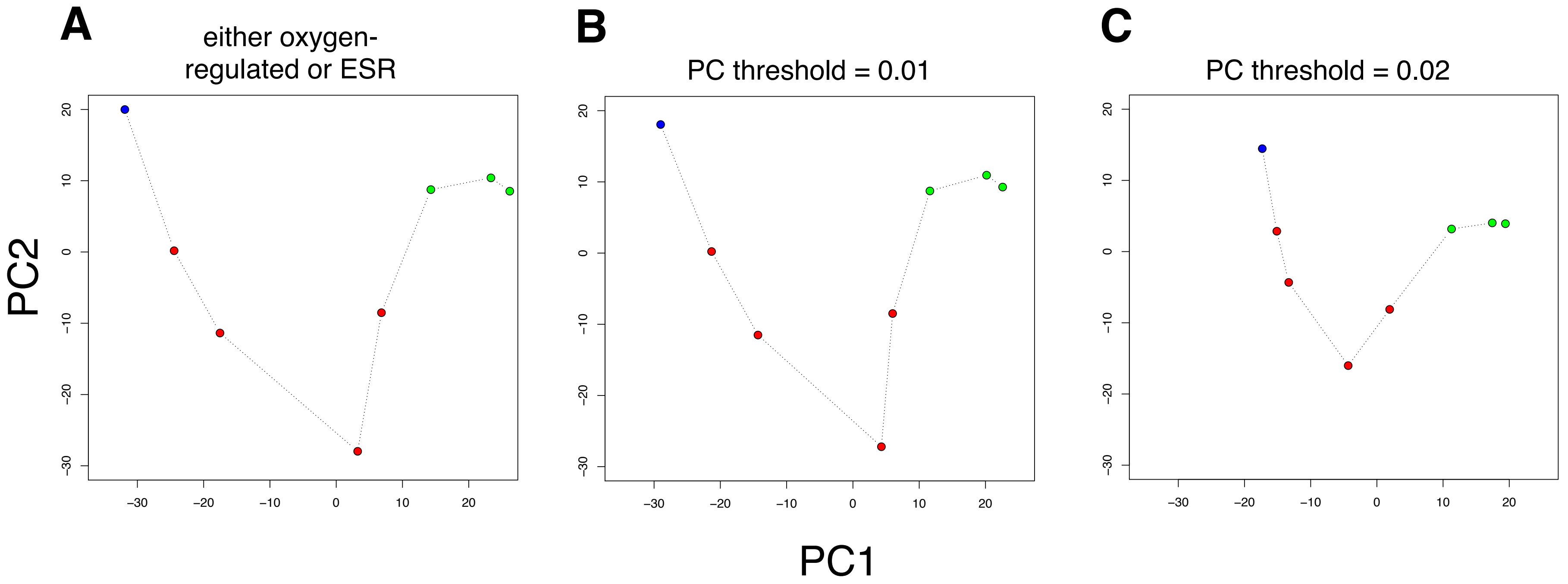

Figure S13. PCA on a subset of genes. **(A)** PCA on the 1705 genes that are either oxygen-regulated or part of the ESR. PC1 and PC2 captured 56.9% and 27.4%, respectively, of the variability in gene expression. **(B)** PCA on the 885 genes that had PC1 and PC2 loadings above 0.01 in the all-gene PCA (Figure 1A). PC1 and PC2 captured 53.5% and 31.8%, respectively, of the variability in gene expression. **(C)** PCA on the 168 genes that had PC1 and PC2 loadings above 0.02 in the all-gene PCA (Figure 1A). PC1 and PC2 captured 65.6% and 25.6%, respectively, of the variability in gene expression.
