# Supplementary material for: Time-Course Analysis of Gene Expression During the Saccharomyces cerevisiae Hypoxic Response
Source: G3 (Bethesda). 2016 Nov 9;7(1):221–31. doi: 10.1534/g3.116.034991 (PMC5217111; doi:10.1534/g3.116.034991)
Supplement: Supplementary file 14 [file 221TableS1.docx]

Table S1. RNA-seq data, statistics and annotations for each gene. The table includes data generated in the current study, or obtained from either the ESR study {Gasch:2000wl} or previous hypoxia microarray studies. The columns are as follows: (A) systematic gene name, (B-I) read counts that have been floored and normalized, (J-K) adjusted p-values for the two statistical tests, (L) the number of previous microarray studies that showed oxygen regulation (numbers calculated in Table S3), (M) whether a gene is present in the environmental stress response (ESR), (N) rationale for removing a gene from further analysis, (O) whether the gene was significantly hypoxic or aerobic (only genes detected by at least one method), and (P) manually-assigned cellular process (only for genes with fold-change ≥ 4). (.xlsx, 971 KB)

Available for download as a .xlsx file at:

http://www.g3journal.org/lookup/suppl/doi:10.1534/g3.116.034991/-/DC1/TableS1.xlsx
