# Supplementary material for: Time-Course Analysis of Gene Expression During the Saccharomyces cerevisiae Hypoxic Response
Source: G3 (Bethesda). 2016 Nov 9;7(1):221–31. doi: 10.1534/g3.116.034991 (PMC5217111; doi:10.1534/g3.116.034991)
Supplement: Supplementary file 16 [file 221TableS2.docx]

Table S2. Sequences and genomic locations of RT-qPCR primers. (.xlsx, 10 KB)

Available for download as a .xlsx file at:

http://www.g3journal.org/lookup/suppl/doi:10.1534/g3.116.034991/-/DC1/TableS2.xlsx
