# Supplementary material for: Time-Course Analysis of Gene Expression During the Saccharomyces cerevisiae Hypoxic Response
Source: G3 (Bethesda). 2016 Nov 9;7(1):221–31. doi: 10.1534/g3.116.034991 (PMC5217111; doi:10.1534/g3.116.034991)
Supplement: Supplementary file 18 [file 221TableS3.docx]

Table S3. The number of previous microarray studies that found a gene to be oxygen-regulated. Eight previous microarray studies {terLinde:1999to, TerLinde:2002gj, Kwast:2002eq, Becerra:2002tf, Lai:2005cq, Lai:2006hd, Hickman:2007br, Hickman:2011iq} were used for this analysis. (.xlsx, 493 KB)

Available for download as a .xlsx file at:

http://www.g3journal.org/lookup/suppl/doi:10.1534/g3.116.034991/-/DC1/TableS3.xlsx
