# Supplementary material for: Time-Course Analysis of Gene Expression During the Saccharomyces cerevisiae Hypoxic Response
Source: G3 (Bethesda). 2016 Nov 9;7(1):221–31. doi: 10.1534/g3.116.034991 (PMC5217111; doi:10.1534/g3.116.034991)
Supplement: Supplementary file 20 [file 221TableS4.docx]

Table S4. Results from GO Slim Mapper at yeastgenome.org. Only shown are the processes that exhibited ≥2-fold enrichment in the regulated gene set compared to the entire genome. (.xlsx, 14 KB)

Available for download as a .xlsx file at:

http://www.g3journal.org/lookup/suppl/doi:10.1534/g3.116.034991/-/DC1/TableS4.xlsx
